# IFN-γ-independent control of *M*. *tuberculosis* requires CD4 T cell-derived GM-CSF and activation of HIF-1α

**DOI:** 10.1371/journal.ppat.1010721

**Published:** 2022-07-25

**Authors:** Erik Van Dis, Douglas M. Fox, Huntly M. Morrison, Daniel M. Fines, Janet Peace Babirye, Lily H. McCann, Sagar Rawal, Jeffery S. Cox, Sarah A. Stanley

**Affiliations:** 1 Department of Molecular and Cell Biology, Division of Immunology and Pathogenesis, University of California, Berkeley, Berkeley, California, United States of America; 2 School of Public Health, Division of Infectious Diseases and Vaccinology, University of California, Berkeley, Berkeley, California, United States of America; Portland VA Medical Center, Oregon Health and Science University, UNITED STATES

## Abstract

The prevailing model of protective immunity to tuberculosis is that CD4 T cells produce the cytokine IFN-γ to activate bactericidal mechanisms in infected macrophages. Although IFN-γ-independent CD4 T cell based control of *M*. *tuberculosis* infection has been demonstrated *in vivo* it is unclear whether CD4 T cells are capable of directly activating macrophages to control infection in the absence of IFN-γ. We developed a co-culture model using CD4 T cells isolated from the lungs of infected mice and *M*. *tuberculosis*-infected murine bone marrow-derived macrophages (BMDMs) to investigate mechanisms of CD4 dependent control of infection. We found that even in the absence of IFN-γ signaling, CD4 T cells drive macrophage activation, M1 polarization, and control of infection. This IFN-γ-independent control of infection requires activation of the transcription factor HIF-1α and a shift to aerobic glycolysis in infected macrophages. While HIF-1α activation following IFN-γ stimulation requires nitric oxide, HIF-1α-mediated control in the absence of IFN-γ is nitric oxide-independent, indicating that distinct pathways can activate HIF-1α during infection. We show that CD4 T cell-derived GM-CSF is required for IFN-γ-independent control in BMDMs, but that recombinant GM-CSF is insufficient to control infection in BMDMs or alveolar macrophages and does not rescue the absence of control by GM-CSF-deficient T cells. In contrast, recombinant GM-CSF controls infection in peritoneal macrophages, induces lipid droplet biogenesis, and also requires HIF-1α for control. These results advance our understanding of CD4 T cell-mediated immunity to *M*. *tuberculosis*, reveal important differences in immune activation of distinct macrophage types, and outline a novel mechanism for the activation of HIF-1α. We establish a previously unknown functional link between GM-CSF and HIF-1α and provide evidence that CD4 T cell-derived GM-CSF is a potent bactericidal effector.

## Author summary

CD4 T cells are essential for immune control of the bacterial pathogen *Mycobacterium tuberculosis*, in part due to CD4 T cell production of the cytokine IFN-γ which activates antimicrobial functions in infected macrophages. Recent evidence points to an IFN-γ-independent role for CD4 T cells during the immune response to *M*. *tuberculosis*, but mechanisms of IFN-γ-independent control have remained elusive. Here we show that, like IFN-γ-mediated control, IFN-γ-independent control requires the transcription factor HIF-1α. HIF-1α is activated via distinct signaling pathways and mediates divergent effects following IFN-γ-mediated and IFN-γ-independent CD4 T cell stimulation, positioning HIF-1α as a critical signaling node for control of *M*. *tuberculosis* in macrophages. Furthermore, we show that CD4 T cell production of the cytokine GM-CSF is required for IFN-γ-independent control and that GM-CSF-mediated control requires HIF-1α. These findings delineate a novel, IFN-γ-independent pathway for CD4 T cell-mediated control that begins with production of GM-CSF and leads to HIF-1α activation to restrict *M*. *tuberculosis* in infected macrophages. Advances in our understanding of CD4 T cell-mediated control of *M*. *tuberculosis* will help guide the development of new and improved vaccines for this important global pathogen.

## Introduction

Increasing our understanding of host immunity to *Mycobacterium tuberculosis* infection has the potential to improve the lives of billions of people around the world, yet major features of the immune response to tuberculosis (TB) remain poorly understood. Broadly, immunity to TB begins with an innate response that induces inflammation and the recruitment of phagocytes, followed by an adaptive response necessary to control infection. A critical aspect of this adaptive immune response is the activation and proliferation of *M*. *tuberculosis* specific CD4 T cells. Mice deficient in CD4 T cells are highly susceptible to TB, and the loss of CD4 T cells in patients suffering from AIDS is strongly correlated with re-activation of dormant *M*. *tuberculosis* infection [1,2]. The cytokine interferon (IFN)-γ is also required for control of TB. Mice deficient for IFN-γ signaling are among the most susceptible strains to *M*. *tuberculosis* infection, and recombinant IFN-γ activates the bactericidal capacity of macrophages [3–6]. The known importance of CD4 T cells and IFN-γ has led to an enduring tenet of TB immunity: that CD4 T cells secrete IFN-γ to control *M*. *tuberculosis* growth in infected macrophages [7].

This basic understanding of protective immunity to TB has come under increasing scrutiny. Comparing different routes of inoculation with the vaccine strain *M*. *bovis* bacille Calmette-Guérin in mice shows that the frequency of IFN-γ-secreting CD4 T cells correlates more closely with disease severity than with protection from TB disease after *M*. *tuberculosis* challenge [8], and human trials with the vaccine candidate MVA85A showed that although this vaccine elicits significant numbers of IFN-γ-secreting CD4 T cells it does not lead to enhanced protection against infection [9,10]. Furthermore, while CD4 T cells are clearly important for TB control in humans, inherited mutations in components of the IFN-γ signaling pathway are not generally associated with susceptibility to *M*. *tuberculosis* but rather with enhanced susceptibility to non-tuberculosis mycobacteria such as *M*. *chelonae*, *M*. *smegmatis*, and *M*. *scrofulaceum* [11]. In mice, adoptive transfer experiments show that IFN-γ-deficient CD4 T cells can control *M*. *tuberculosis in vivo* [1,12,13] with IFN-γ production accounting for only 30% of CD4 T cell-mediated control in the lungs [13]. Collectively, these findings point to an important unknown mechanism of control mediated by CD4 T cells that is independent of IFN-γ.

Immune resistance to TB requires a fine balance between pro-inflammatory effectors that control bacterial replication and anti-inflammatory immune regulation that prevents immunopathology. CD4 T cells themselves contribute to both arms, secreting pro-inflammatory molecules such as IFN-γ and TNFα and anti-inflammatory cytokines including IL-10 and TGF-β, complicating the interpretation of *in vivo* experiments. Whether control by IFN-γ-deficient CD4 T cells following adoptive transfer is due to immunoregulatory effects or to the antibacterial effects of a specific IFN-γ-independent effector remains an open question. Thus far, adoptive transfer experiments have precluded a role for CD4 T cell expression of TNFα, Fas and perforin in IFN-γ-independent control [12], and have not demonstrated that a CD4 T cell-derived IFN-γ-independent effector can stimulate cell-intrinsic control of bacterial replication.

Recently, the cytokine granulocyte-macrophage colony-stimulating factor (GM-CSF) was shown to have a potential role in CD4 T cell-mediated control of TB. Recombinant GM-CSF (rGM-CSF) controls *M*. *tuberculosis* growth in mouse peritoneal macrophages and human monocytes, and GM-CSF-deficient (*Csf2*^*-/-*^) mice have significantly higher bacterial burden in the lungs compared to wild-type and succumb more rapidly to infection [14–16]. However, *Csf2*^*-/-*^ mice and humans with inherited mutations in the GM-CSF receptor have a defect in alveolar macrophage development which complicates the interpretation of whole-animal knockout experiments [17,18]. Finally, adoptive transfer experiments show that *Csf2*^*-/-*^ CD4 T cells only exhibit less control than wild-type when transferred into mice deficient for GM-CSF [15], so it remains unclear whether CD4 T cell production of GM-CSF induces macrophage-intrinsic control of *M*. *tuberculosis*.

In this study, we use an *in vitro* co-culture system to determine whether an IFN-γ-independent CD4 T cell effector can induce cell intrinsic control of *M*. *tuberculosis* replication. We show that lung-derived CD4 T cells and multiple *in vitro*-differentiated T cell subsets exhibit IFN-γ-independent control in infected macrophages via a secreted and proteinaceous effector, and we use RNA sequencing and cytokine profiling to investigate possible mechanisms. Like IFN-γ-mediated control, IFN-γ-independent control by CD4 T cells requires the transcription factor hypoxia inducible factor-1α (HIF-1α), activation of which leads to a metabolic switch to aerobic glycolysis and the formation of macrophage lipid droplets (LDs). These changes occur independent of the IFN-γ-induced second messenger nitric oxide (NO)—normally required for HIF-1α activation during *M*. *tuberculosis* infection—indicating a novel mechanism of HIF-1α activation. Furthermore, we show that there is no role for CD4 T cell production of TNFα, Type I IFN, CD153 (*Tnfsf8*) or CD40, but that CD4 T cell secretion of GM-CSF is required for IFN-γ-independent control. rGM-CSF is sufficient to control *M*. *tuberculosis* in mouse peritoneal macrophages, requires HIF-1α for control and correlates with the production of macrophage LDs. However, rGM-CSF is insufficient for control in bone marrow-derived macrophages (BMDMs) or alveolar macrophages and, surprisingly, does not rescue the loss of IFN-γ-independent control by GM-CSF-deficient CD4 T cells, indicating either differences in recombinant and CD4 T cell-derived GM-CSF function or an unknown second CD4 T cell effector necessary for GM-CSF-mediated control in BMDMs. These results advance our understanding of CD4 T cell-mediated control of *M*. *tuberculosis*, establish CD4 T cell-derived GM-CSF as a potent bactericidal effector and, by uncovering a novel mechanism for HIF-1⍺ activation, emphasize the central importance of HIF-1⍺ for intracellular immunity to TB.

## Results

### Lung-derived and *in vitro*-differentiated CD4 T cells exhibit IFN-γ-independent control of *M*. *tuberculosis*

**Fig 1:**
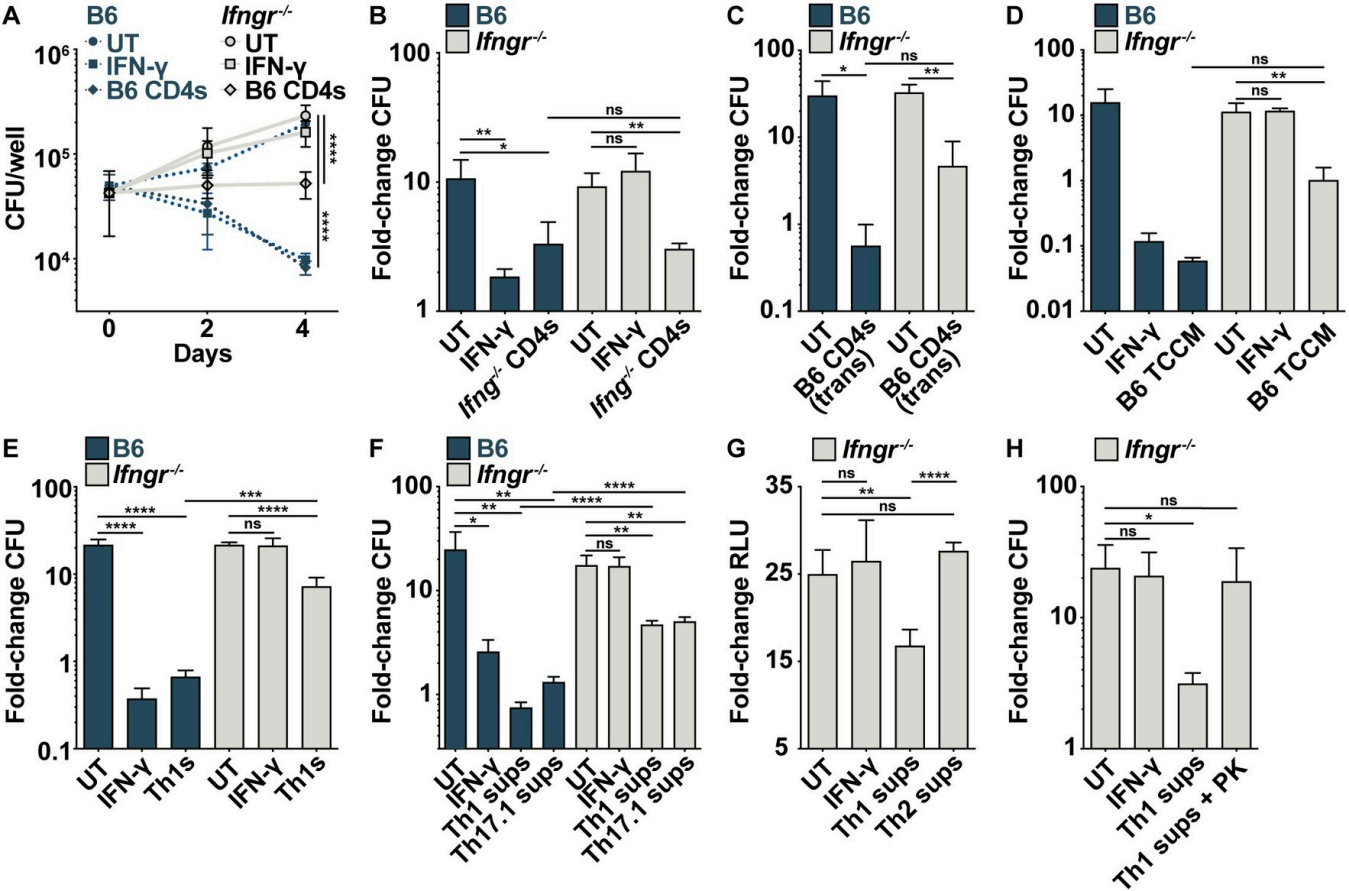
Lung-derived and *in vitro*-differentiated CD4 T cells control *M*. *tuberculosis* growth independent of IFN-γ. (**A**) CFU/well at d 0, 2 and 4 postinfection for wild-type and *Ifngr*^*-/-*^ BMDMs co-cultured with lung-derived CD4 T cells. (**B**) CFU fold-change at d 5 postinfection for wild-type and *Ifngr*^*-/-*^ BMDMs co-cultured with lung-derived *Ifng*^*-/-*^ CD4 T cells. (**C**) CFU fold-change at d 4 postinfection for wild-type and *Ifngr*^*-/-*^ BMDMs cultured with lung-derived CD4 T cells in transwells. (**D**) CFU fold-change at d 5 postinfection for wild-type and *Ifngr*^*-/-*^ BMDMs treated with conditioned media from lung-derived CD4 T cells (T cell conditioned media, TCCM). (**E**) CFU fold-change at d 5 postinfection for wild-type and *Ifngr*^*-/-*^ BMDMs co-cultured with *in vitro* differentiated Th1 cells. (**F**) CFU fold-change at d 4 postinfection for wild-type and *Ifngr*^*-/-*^ BMDMs treated with Th1 or Th17.1 supernatants (sups). (**G**) RLU fold-change at d 5 postinfection for *Ifngr*^*-/-*^ BMDMs treated with Th1 or Th2 sups. (**H**) CFU fold-change at d 5 postinfection for *Ifngr*^*-/-*^ BMDMs treated with Th1 sups +/- Proteinase K (PK). Figures are representative of two (H) or three or more (A)-(G) experiments. Error bars are SD from four (A)-(B), (D)-(H) or three (C) replicate samples, *p<0.05, **p<0.01, ***p<0.001, ****p<0.0001 by unpaired t-test.

To confirm that CD4 T cells can control *M*. *tuberculosis* independent of IFN-γ, we cultured CD4 T cells derived from the lungs of infected mice with infected *Ifngr*^*-/-*^ BMDMs and observed IFN-γ-independent control of bacterial growth (Fig 1A). This control was dose dependent in *Ifngr*^*-/-*^ BMDMs as increasing the ratio of T cells to macrophages led to increased bacterial control (S1A and S1B Fig). Similarly, CD4 T cells from the lungs of infected *Ifng*^*-/-*^ mice controlled bacterial growth during co-culture with wild-type BMDMs (Fig 1B). Conditioned media from CD4 T cells controlled growth in *Ifngr*^*-/-*^ BMDMs, as did CD4 T cells in transwells, showing that IFN-γ-independent control is mediated by a secreted effector (Fig 1C and 1D). Since the bulk CD4+ population of cells from the lungs will include many T cell subsets, including unconventional T cells, we next asked whether a specific T cell subset is sufficient for IFN-γ-independent control by *in vitro* differentiating Th1, Th2 and Th17.1 T cells using CD4+ T cells from *M*. *tuberculosis*-specific TCR transgenic mice. Th1s restricted bacterial growth during co-culture with *Ifngr*^*-/-*^ BMDMs (Fig 1E), and supernatants from Th1s exhibited dose-dependent IFN-γ-independent control with no impact on cell viability (Figs 1F and S1D and S1E). Supernatants from Th17.1 T cells, which are RORγt+ and co-express IFN-γ and IL-17 (S1C Fig) [19], also demonstrated IFN-γ-independent control while supernatants from Th2s did not (Fig 1F and 1G). Taken together, these results show that lung-derived CD4 T cells and *in vitro* differentiated Th1 and Th17.1 cells secrete an IFN-γ-independent effector that induces cell-intrinsic control of *M*. *tuberculosis*. To test whether this effector is amino acid-based, we treated Th1 supernatants with proteinase K and observed a loss of IFN-γ-independent control (Fig 1H). Thus, a proteinaceous effector secreted by CD4 T cells activates BMDMs to control *M*. *tuberculosis* independent of IFN-γ.

### IFN-γ is not required for T cell-mediated macrophage activation and polarization during *M*. *tuberculosis* infection

**Fig 2:**
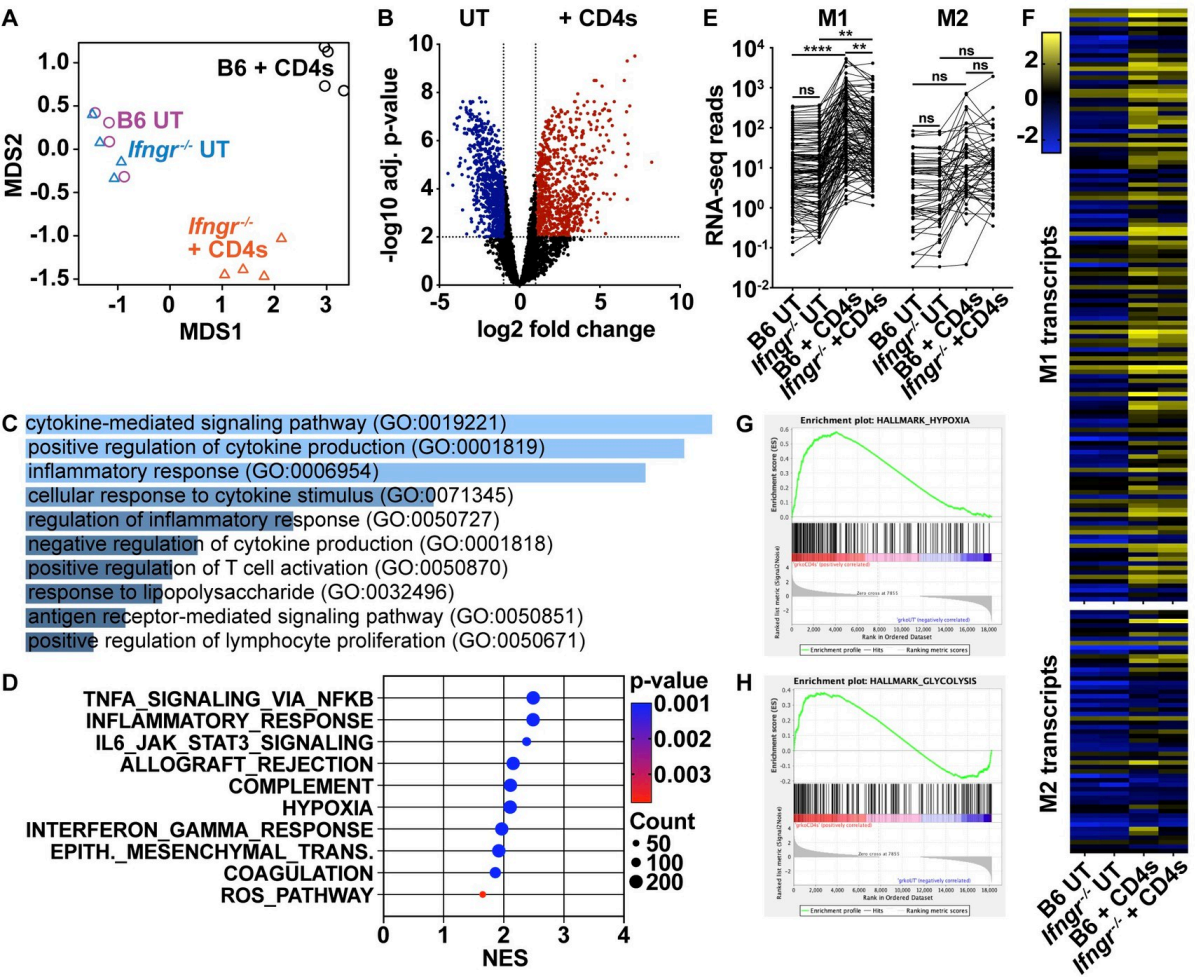
IFN-γ is not required for T cell-mediated macrophage activation and polarization during *M*. *tuberculosis* infection. (**A**)-(**B**) RNA-seq at 24 h postinfection for wild-type and *Ifngr*^*-/-*^ BMDMs co-cultured with lung-derived CD4 T cells. (A) Principal component analysis plot, (B) Volcano plot of genes in *Ifngr*^*-/-*^ BMDMs with >2-fold change in gene expression and adj. p-value >0.05 following cell co-culture. (**C**) Top ten enriched ontology clusters from Ingenuity Pathway Analysis of genes with >2-fold upregulation in both wild-type and *Ifngr*^*-/-*^ BMDMs following co-culture. (**D**) Top ten enriched gene sets from the MSigDB H: Hallmark collection from *Ifngr*^*-/-*^ BMDMs following co-culture. (**E**)-(**F**) RNA-seq reads (E) and heat map (F) of M1- or M2-associated transcripts. (**G**)-(**H**) Gene Set Enrichment Analysis plots of (G) Hypoxia or (H) Glycolysis gene sets in *Ifngr*^*-/-*^ BMDMs following co-culture. Figures represent data from four replicate experiments, **p<0.01, ****p<0.0001 by Tukey post hoc test.

To identify IFN-γ-independent signaling pathways activated by CD4 T cells, we performed RNA sequencing on *M*. *tuberculosis*-infected wild-type and *Ifngr*^*-/-*^ BMDMs after 24 hours of co-culture (Fig 2A). CD4 T cells induced differential regulation of 1825 genes in wild-type BMDMs and 1142 genes in *Ifngr*^*-/-*^ BMDMs compared to untreated (Fig 2B) (S1 Table). Although wild-type and *Ifngr*^*-/-*^ BMDMs infected with *M*. *tuberculosis* were transcriptionally very similar prior to activation, the transcriptome of these genotypes of macrophages diverged after CD4 T cell co-culture (Fig 2A). This is likely due in part to the presence or absence of IFN-γ signaling as IFN-γ alone regulates the expression of >2500 genes during *M*. *tuberculosis* infection [20]. Still, 769 genes were altered in an IFN-γ-independent manner, with >2-fold upregulation in both wild-type and *Ifngr*^*-/-*^ BMDMs (S1 Table). Gene ontology (GO) analysis of these transcripts revealed cytokine-mediated signaling, positive regulation of cytokine production, and inflammatory responses as the top three cellular processes mediated by CD4 T cells independent of IFN-γ (Fig 2C) [21–23]. Gene Set Enrichment Analysis (GSEA) from co-cultured *Ifngr*^*-/-*^ BMDMs supported these findings, with TNFα Signaling via NF-κB (FDR < 0.001) and Inflammatory Response (FDR < 0.001) as the top two enriched Hallmark gene sets from the Molecular Signatures Database (MSigDB) (Fig 2D). Further analysis using the MsigDB Curated gene sets revealed an enrichment of Cytokine-Cytokine Receptor Interaction (FDR < 0.001), GM-CSF Pathway (FDR < 0.05), HIF-1 TF Pathway (FDR < 0.05), and Prostaglandin Signaling (FDR < 0.01) in *Ifngr*^*-/-*^ BMDMs following CD4 T cell co-culture (S2A Fig). Gene sets from the MSigDB Curated collection that were significantly altered in wild-type or *Ifngr*^*-/-*^ BMDMs following CD4 T cell co-culture or in wild-type BMDMs treated with IFN-γ were visualized in a network enrichment map (S2B Fig). This showed that a majority of gene sets relating to NF-κB signaling, interleukin signaling, GPCR signaling, the inflammatory response, and immune signaling and antigen presentation were enriched in all three conditions. Similarly, most gene sets relating to the DNA damage response, mRNA processing, meiosis, mitosis, and SUMOylation and nuclear transport were negatively enriched in all three conditions. Network enrichment analysis revealed only small differences between treatment conditions at the level of cellular function (S2B Fig). Thus, transcriptional differences attributable to the presence or absence of IFN-γ signaling seen Fig 2A do not reflect major changes in cellular processes mediated by CD4 T cells that may be involved in control of infection.

We next determined the state of macrophage polarization after CD4 T cell co-culture using a published dataset of genes that correspond to either classically activated (M1) or alternatively activated (M2) macrophages [24]. We found no difference in macrophage polarization between untreated *M*. *tuberculosis*-infected wild-type and *Ifngr*^*-/-*^ BMDMs, and no significant increase in the expression of genes associated with M2 macrophages after CD4 T cell co-culture in either genotype (Fig 2E). However, there was significant upregulation of genes associated with M1 macrophages after CD4 T cell co-culture in both genotypes, with wild-type BMDMs slightly more polarized (Fig 2E and 2F). Finally, GSEA revealed an enrichment of transcripts for the M1-associated gene sets Hypoxia (FDR < 0.001) and Glycolysis (FDR < 0.05) in *Ifngr*^*-/-*^ BMDMs during co-culture (Fig 2G and 2H). Collectively, these results show comparable patterns of activation in wild-type and *Ifngr*^*-/-*^ macrophage during CD4 T cell co-culture, indicating that CD4 T cells elicit significant polarizing, inflammatory and antimicrobial effects in *M*. *tuberculosis*-infected macrophages irrespective of IFN-γ signaling.

### IFN-γ-independent control requires an NO-independent HIF-1α-mediated shift to aerobic glycolysis

**Fig 3:**
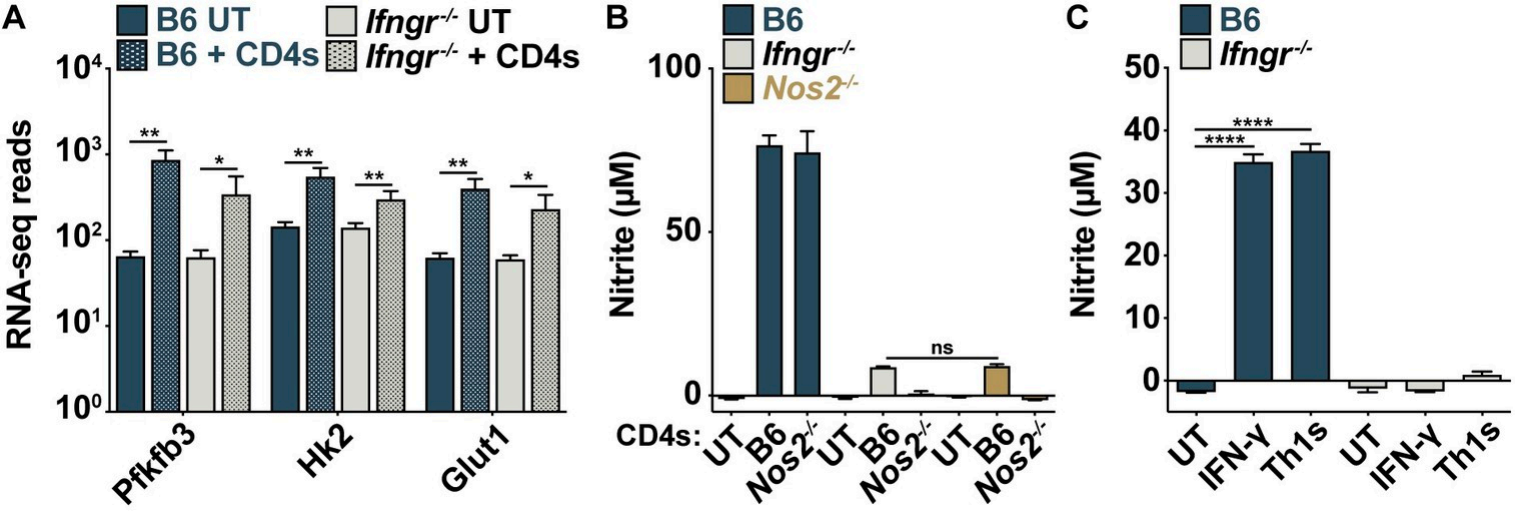
CD4 T cells induce IFN-γ- and NO-independent increases in glycolytic gene expression. (**A**) RNA-seq reads of glycolytic genes. (**B**) Griess assay at 48 h postinfection for wild-type, *Ifngr*^*-/-*^, and *Nos2*^*-/-*^ BMDMs co-cultured with a 10:1 ratio of wild-type or *Nos2*^*-/-*^ lung-derived CD4 T cells to macrophages. (**C**) Griess assay at 48 h postinfection for wild-type and *Ifngr*^*-/-*^ BMDMs co-cultured with Th1 cells. Figures represent data from four replicate experiments (A) or are representative of two (B) or three (C) independent experiments. Error bars are SD from four independent experiments (A) or four replicate samples (B)-(C), *p<0.05, **p<0.01, ****p<0.0001 by unpaired t-test.

Classically activated macrophages rely on HIF-1α-induced glycolysis to maintain an activated and polarized phenotype [25]. During *M*. *tuberculosis* infection, IFN-γ is sufficient to induce a HIF-1α-mediated metabolic switch to aerobic glycolysis that is required for IFN-γ-mediated control [20]. CD4 T cells also induced significant upregulation of genes associated with aerobic glycolysis and an increase in glucose uptake, both with and without IFN-γ signaling (Figs 3A and S3A). Nitric oxide (NO) production by inducible nitric oxide synthase (iNOS) is required for IFN-γ-mediated HIF-1α stabilization and the shift to aerobic glycolysis, and iNOS-deficient (*Nos2*^*-/-*^) BMDMs have a defect in cell intrinsic control of *M*. *tuberculosis* following IFN-γ signaling [26]. To explore a role for NO in IFN-γ-independent control, we first asked whether NO is produced during CD4 T cell co-cultures. While NO is produced in abundance by IFN-γ-activated BMDMs during *M*. *tuberculosis* infection, there is little to no NO production induced by CD4 T cells or Th1 supernatants in the absence of IFN-γ signaling (Fig 3B and 3C). A low level of NO was detected during CD4 T cell co-cultures only when CD4 T cells themselves expressed iNOS, but this amount of CD4 T cell-derived NO does not contribute to control of bacterial growth in macrophages (Figs 3B and S3B). Therefore, IFN-γ-independent control by lung CD4 T cells and *in vitro* differentiated Th1s is independent of NO.

**Fig 4:**
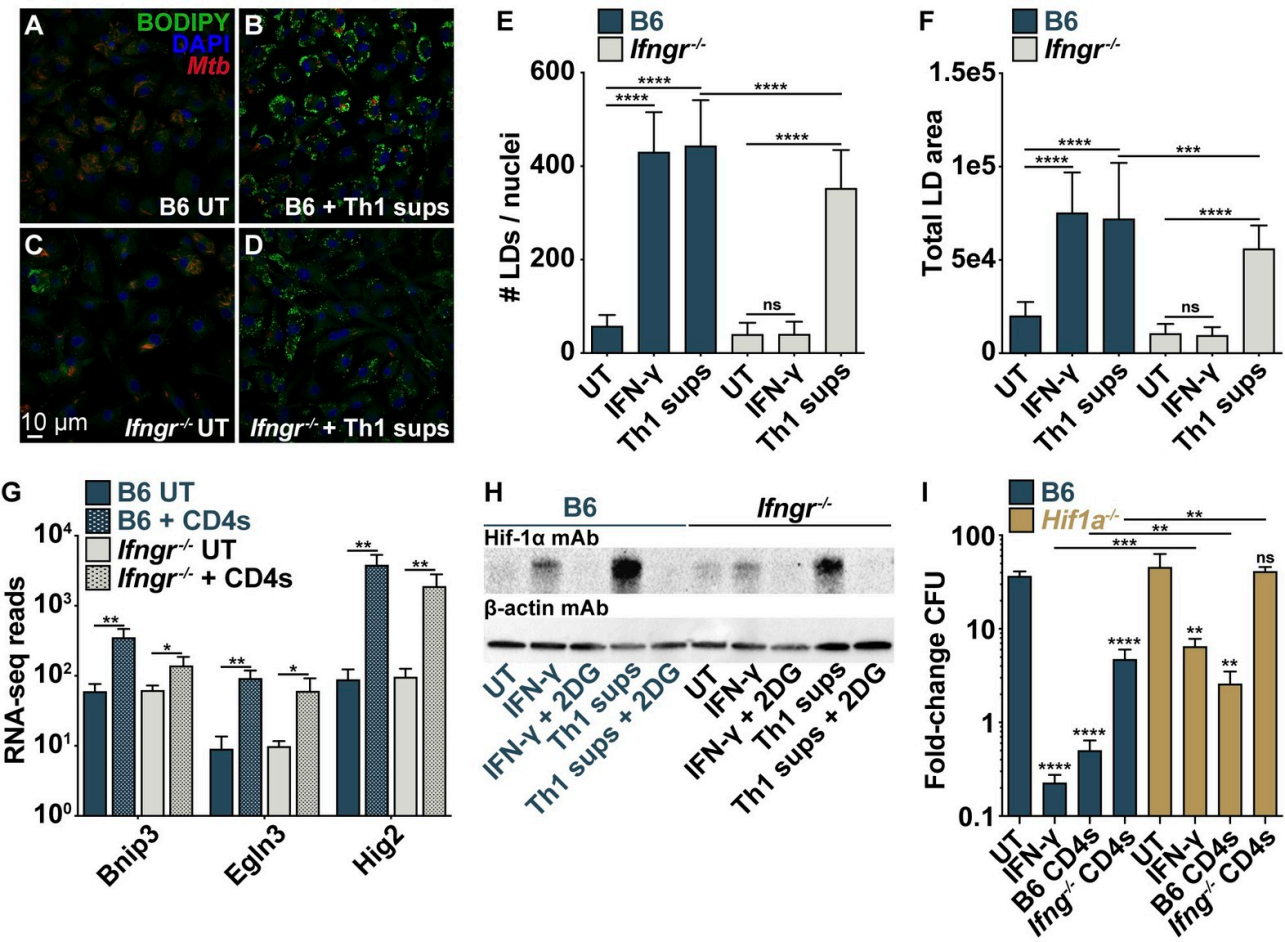
IFN-γ-independent control requires the transcription factor HIF-1α. (**A**)-(**D**) Microscopy for host lipid droplets (LDs) at d 3 postinfection for wild-type and *Ifngr*^*-/-*^ BMDMs treated with Th1 supernatants (sups). (**E**)-(**F**) Quantification of (A)-(D) for (E) average number of LDs per BMDM and (F) total LD area. (**G**) RNA-seq reads of HIF-1α target genes. (**H**) Western blot for HIF-1α on cell lysates 12 h postinfection for wild-type and *Ifngr*^*-/-*^ BMDMs treated with Th1 sups and 2-DG. (**I**) CFU fold-change at d 5 postinfection for wild-type and *Hif1a*^*-/-*^ BMDMs co-cultured with wild-type or *Ifng*^*-/-*^ lung-derived CD4 T cells. Figures represent data from four replicate experiments (G) or are representative of two (H) or three (A)-(F), (I) independent experiments. Error bars are SD from four independent experiments (G), four replicate samples (I), or 48 images from four replicate wells (E)-(F) *p<0.05, **p<0.01, ***p<0.001, ****p<0.0001 by unpaired t-test; p-values above bars are relative to UT for each genotype.

IFN-γ signaling stabilizes and activates the transcription factor HIF-1α during *M*. *tuberculosis* infection and HIF-1α-deficient (*Hif1a*^*-/-*^) BMDMs have a defect in IFN-γ-mediated control [20]. NO helps stabilize HIF-1α and the two form a positive feedback loop that is necessary for the antibacterial effect of IFN-γ [26]. Activation of HIF-1α also mediates lipid droplet (LD) biogenesis in IFN-γ-stimulated BMDMs which supports host immunity by serving as a platform for eicosanoid production [27]. Despite an absence of NO and IFN-γ signaling, we observed an increase in the number and size of LDs in infected wild-type and *Ifngr*^*-/-*^ BMDMs after treatment with Th1 supernatants (Fig 4A–4F), suggesting a role for HIF-1α in IFN-γ-independent control. We also observed significant upregulation of multiple HIF-1α target genes in *Ifngr*^*-/-*^ BMDMs during CD4 T cell co-culture (Fig 4G), and bioinformatic analysis revealed an overrepresentation of genes with predicted HIF-1α binding sites in *Ifngr*^*-/-*^ BMDMs (S2A and S3C Figs). We then treated *Ifngr*^*-/-*^ BMDMs with Th1 supernatants and observed an increase in HIF-1α protein by western blot (Fig 4H). As has been demonstrated for IFN-γ-mediated HIF-1α stabilization [20], this was dependent on a metabolic switch to glycolysis since treatment with the glycolysis inhibitor 2-deoxy-D-glucose (2-DG) reversed HIF-1α stabilization by Th1 supernatants (Fig 4H).

To determine whether HIF-1α is required for IFN-γ-independent control of infection we co-cultured *Ifng*^*-/-*^ CD4 T cells with wild-type or *Hif1a*^*-/-*^ BMDMs. As expected, *Hif1a*^*-/-*^ BMDMs had significantly less control than wild-type BMDMs following IFN-γ stimulation (Fig 4I) [20]. Furthermore, while *Ifng*^*-/-*^ CD4 T cells controlled bacterial growth in wild-type BMDMs, we observed no control of bacterial growth in *Hif1a*^*-/-*^ BMDMs (Fig 4I), and 2-DG partially reduced IFN-γ-independent control by Th1 supernatants (S3D Fig). These results demonstrate that HIF-1α stabilization and aerobic glycolysis are necessary for IFN-γ-independent control of *M*. *tuberculosis* by CD4 T cells. The observation that HIF-1α activation and LD accumulation occur independent of IFN-γ and NO during *M*. *tuberculosis* infection, and that HIF-1α is required for IFN-γ-independent control, solidifies HIF-1α as a critical signaling node after CD4 T cell activation of infected macrophages and suggests that pathways of IFN-γ-dependent and IFN-γ-independent control by CD4 T cells converge on HIF-1α activation.

### CD4 T cell-derived GM-CSF is necessary for IFN-γ-independent control of *M*. *tuberculosis*

**Fig 5:**
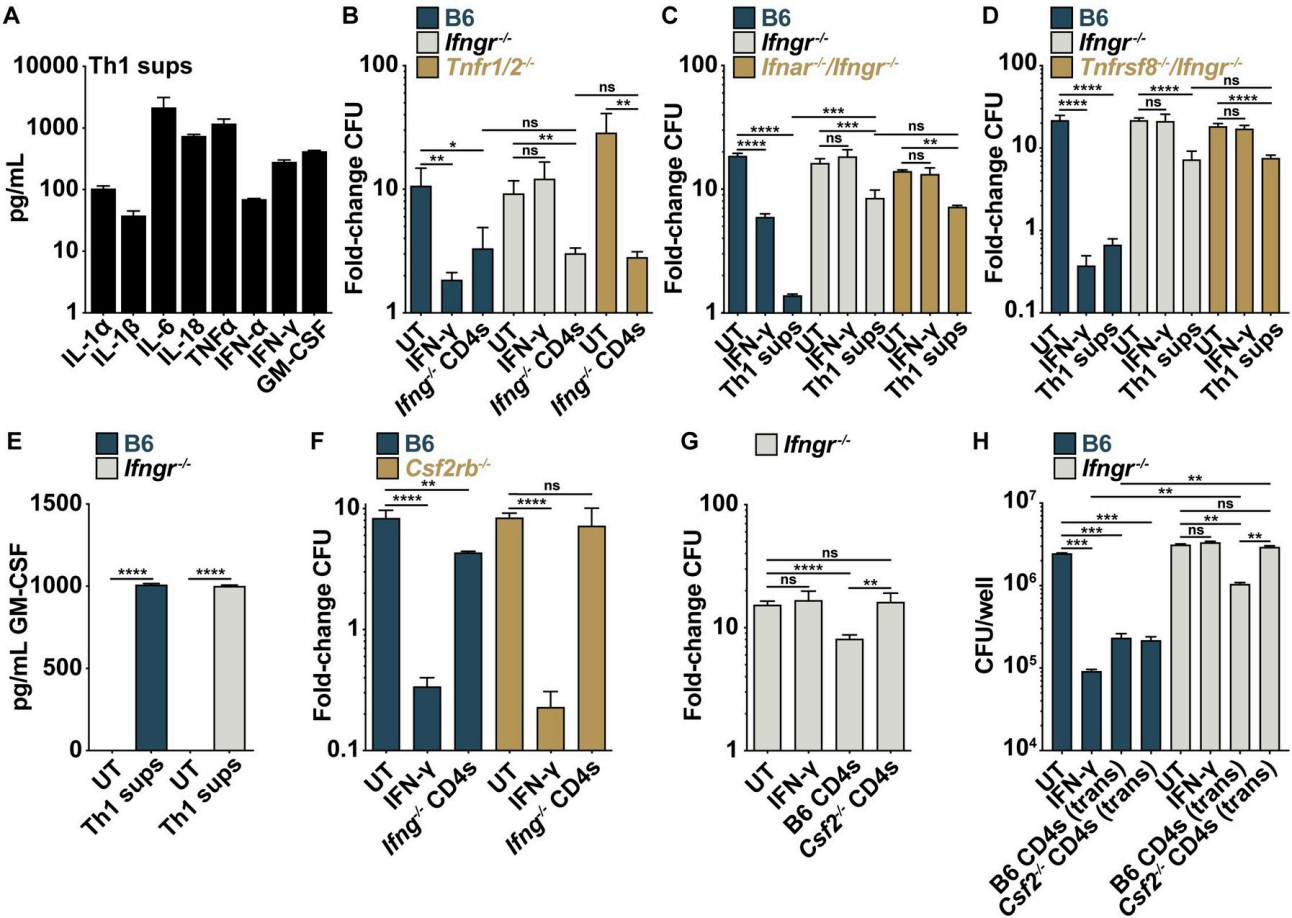
IFN-γ-independent control of *M*. *tuberculosis* requires CD4 T cell-derived GM-CSF. (**A**) Concentration of select cytokines in *in vitro* differentiated Th1 supernatants (sups). (**B**) CFU fold-change at d 5 postinfection for wild-type, *Ifngr*^*-/-*^, and *Tnfr1/2*^*-/-*^ BMDMs co-cultured with *Ifng*^*-/-*^ lung-derived CD4 T cells. (**C**) CFU fold-change at d 5 postinfection for wild-type, *Ifngr*^*-/-*^, and *Ifnar*^*-/-*^/*Ifngr*^*-/-*^ double-knockout BMDMs treated with Th1 sups. (**D**) CFU fold-change at d 5 postinfection for wild-type, *Ifngr*^*-/-*^ and *Tnfrsf8*^*-/-*^/*Ifngr*^*-/-*^ double-knockout BMDMs treated with Th1 sups. (**E**) ELISA for GM-CSF concentration at 24 h postinfection in wild-type and *Ifngr*^*-/-*^ BMDMs treated with Th1 sups. (**F**) CFU fold-change at d 5 postinfection for wild-type and *Csf2rb*^*-/-*^ BMDMs co-cultured with *Ifng*^*-/-*^ lung-derived CD4 T cells. (**G**) CFU fold-change at d 5 postinfection for *Ifngr*^*-/-*^ BMDMs co-cultured with wild-type or *Csf2*^*-/-*^ lung-derived CD4 T cells. (**H**) CFU-fold change at d 4 postinfection for wild-type and *Ifngr*^*-/-*^ BMDMs cultured with wild-type or *Csf2*^*-/-*^ lung-derived CD4 T cells in transwells. Figures represent seven (A) or three (E) biological replicates or are representative of two (B), (D), (H) or at least three (C), (F)-(G) independent experiments. Error bars are SD from seven (A) or three (B) biological replicates or four (B)-(D), (F)-(G) or three (H) replicate samples, *p<0.05, **p<0.01, ***p<0.001, ****p<0.0001 by unpaired t-test.

To determine which secreted effector is necessary for IFN-γ-independent control, we performed cytokine profiling of Th1 supernatants (Fig 5A) and identified multiple inflammatory cytokines previously implicated in immunity to TB including TNFα, IFN-α, IL-1β, IFN-γ, and GM-CSF. *Ifng*^*-/-*^ CD4 T cells had no loss of control in co-culture with *Tnfr1*^*-/-*^*/Tnfr2*^*-/-*^ BMDMs compared to wild-type (Fig 5B), and Th1 supernatants had no loss of control in *Ifnar*^*-/-*^*/Ifngr*^*-/-*^ BMDMs compared to *Ifngr*^*-/-*^ BMDMs (Fig 5C). Additionally, blocking antibodies for CD40 had no effect on IFN-γ-independent control by lung CD4 T cells (S4A Fig). Thus, IFN-γ-independent CD4 T cell control of *M*. *tuberculosis* in BMDMs does not require TNFα, Type I IFN, or CD40. The CD4 T cell surface molecule CD30 ligand (*Tnfsf8*) has been implicated in IFN-γ-independent control of *M*. *tuberculosis in vivo* [28]. However, we observed little to no expression of the gene for CD30 (*Tnfrsf8*) in BMDMs in the presence or absence of CD4 T cells (S4B Fig) and no loss of IFN-γ-independent control in BMDMs doubly deficient for CD30 and IFNγR (*Tnfrsf8*^*-/-*^*/Ifngr*^*-/-*^) compared to *Ifngr*^*-/-*^ BMDMs alone (Fig 5D). CD30 and CD30 ligand are cell-surface molecules so, together with the observation that IFN-γ-independent control does not require cell-to-cell contact (Figs 1C and 1D and 1F), these results argue that the role of CD30 ligand on CD4 T cells during TB infection is likely immunoregulatory rather than directly bactericidal.

We next tested a role for the cytokine GM-CSF in IFN-γ-independent control of *M*. *tuberculosis*. GM-CSF is secreted by Th1 and Th17.1 T cells (S5C Fig) and can be found in the supernatant of BMDMs treated with Th1 supernatants or co-cultured with CD4 T cells from the lungs of wild-type or *Ifng*^*-/-*^ mice (Figs 5E and S4D). *Csf2ra* and *Csf2rb*, genes for the two GM-CSF receptor subunits, are expressed in BMDMs and transcription increases more than 3-fold following CD4 co-culture (S4E and S4F Fig), and GSEA using the MSigDB C3 Transcription Factor Target Prediction gene set revealed that the set of target genes for the transcription factor STAT5, which helps mediate GM-CSF receptor signaling, is significantly enriched in *Ifngr*^*-/-*^ BMDMs during CD4 T cell co-culture (FDR < 0.001) (S4G Fig). Importantly, while *Ifng*^*-/-*^ CD4s controlled *M*. *tuberculosis* in wild-type BMDMs, this control was lost in BMDMs lacking the GM-CSF receptor (*Csf2rb*^*-/-*^) (Fig 5F). Furthermore, wild-type but not GM-CSF deficient (*Csf2*^*-/-*^) CD4 T cells controlled bacterial growth during co-culture or when cultured in transwells with *Ifngr*^*-/-*^ BMDMs (Fig 5G and 5H). Taken together, these results show that lung CD4 T cells secrete GM-CSF to control *M*. *tuberculosis* growth in macrophages independent of IFN-γ.

### GM-CSF activates HIF-1α to restrict *M*. *tuberculosis* in peritoneal macrophages

**Fig 6:**
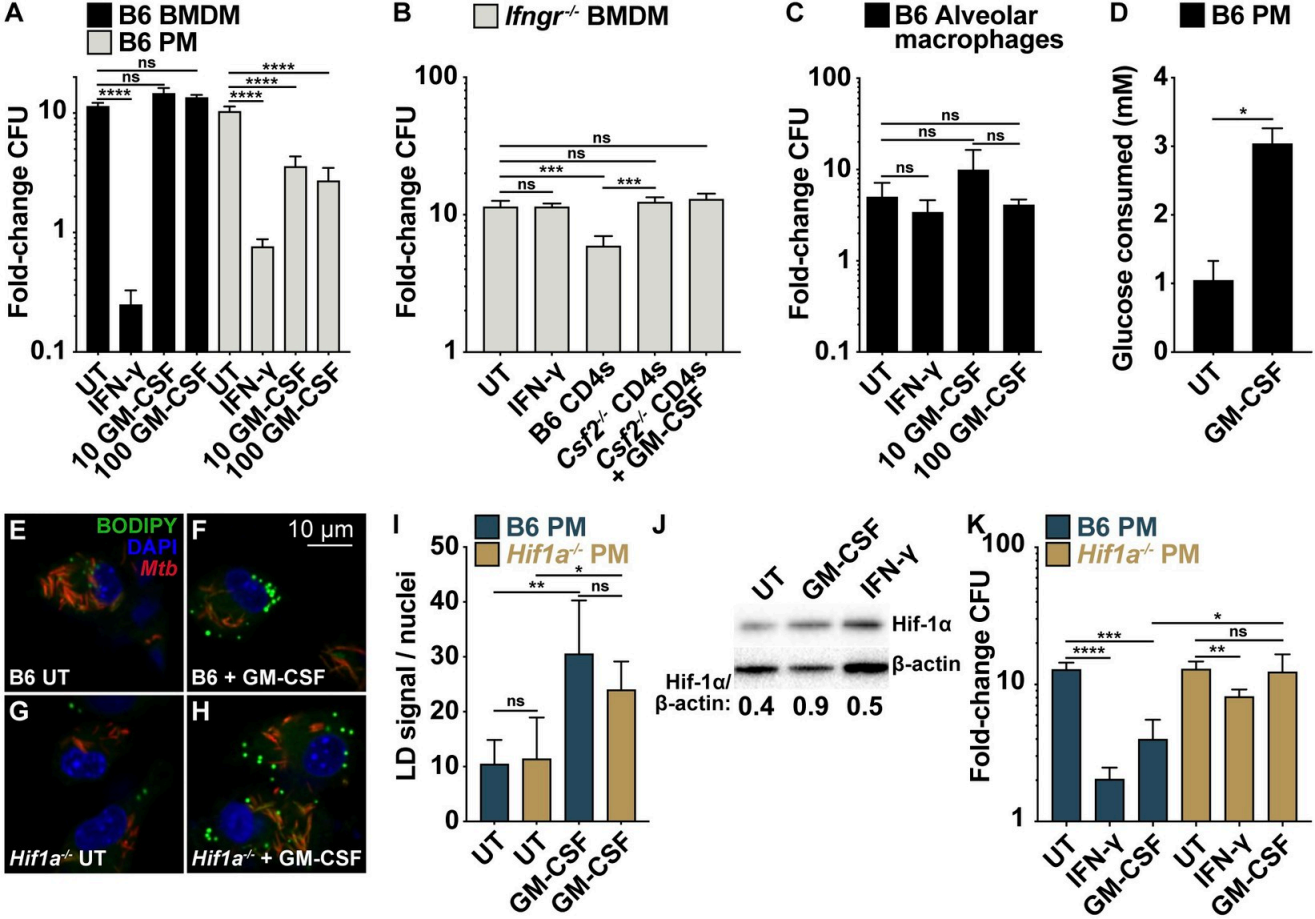
GM-CSF activates HIF-1α to restrict *M*. *tuberculosis* in peritoneal macrophages. (**A**) CFU fold-change at d 4 postinfection for BMDMs and peritoneal macrophages treated with IFN-γ or 10 or 100 ng/mL GM-CSF. (**B**) CFU fold-change at d 5 postinfection for *Ifngr*^*-/-*^ BMDMs co-cultured with wild-type or *Csf2*^*-/-*^ lung CD4 T cells and GM-CSF. (**C**) CFU fold-change at d 4 postinfection for alveolar macrophages treated with GM-CSF. (**D**) Glucose consumption at 24 h postinfection for peritoneal macrophages treated with GM-CSF. (**E**)-(**H**) Microscopy for host lipid droplets (LDs) at 24 h postinfection for wild-type or *Hif1a*^*-/-*^ peritoneal macrophages treated with GM-CSF. (**I**) Quantification of (E)-(H) for LD signal per macrophage. (**J**) Western blot for HIF-1α on cell lysates 20 h postinfection for peritoneal macrophages treated with GM-CSF. HIF-1α/β-actin ratio is indicated and was quantified using ImageJ. (**K**) CFU fold-change at d 4 postinfection for wild-type and *Hif1a*^*-/-*^ peritoneal macrophages treated with GM-CSF. Figures are representative of two (E)-(J) or three or more (A)-(D), (K) experiments. Error bars are SD from four replicate samples (A)-(D), (K) or four replicate wells (I), *p<0.05, **p<0.01, ***p<0.001, ****p<0.0001 by unpaired t-test.

As reported, recombinant GM-CSF (rGM-CSF) is sufficient to control *M*. *tuberculosis* growth in peritoneal macrophages (Fig 6A) [15,29]. However, despite a clear requirement for GM-CSF signaling in BMDMs during CD4 T cell co-culture, rGM-CSF was not sufficient to induce control of *M*. *tuberculosis* in BMDMs even at high concentrations and regardless of the biological source of recombinant protein (Figs 6A and S5A). Moreover, rGM-CSF from multiple sources did not rescue the loss of control by *Csf2*^*-/-*^ CD4 T cells in co-culture with *Ifngr*^*-/-*^ BMDMs (Figs 6B and S5B). Thus, while GM-CSF is sufficient to induce control of *M*. *tuberculosis* in peritoneal macrophages, it is necessary but not sufficient for CD4 T cell-mediated control in BMDMs, reflecting either differences in the ability of CD4 T cell-derived and recombinant GM-CSF to effectively signal to BMDMs or secondary defects in *Csf2*^*-/-*^ CD4 T cells required for IFN-γ-independent control. Finally, rGM-CSF did not control bacterial growth in primary-isolated alveolar macrophages (Fig 6C), which is consistent with previous literature demonstrating that alveolar macrophages downregulate the GM-CSF Receptor-specific subunit CSF2Rα upon *M*. *tuberculosis* infection *in vivo* [30]. Collectively, these findings suggest major differences between these cell types and indicate that there are important differences in the ability of diverse macrophage subsets to control *M*. *tuberculosis* in response to the same immune stimulus.

**Fig 7:**
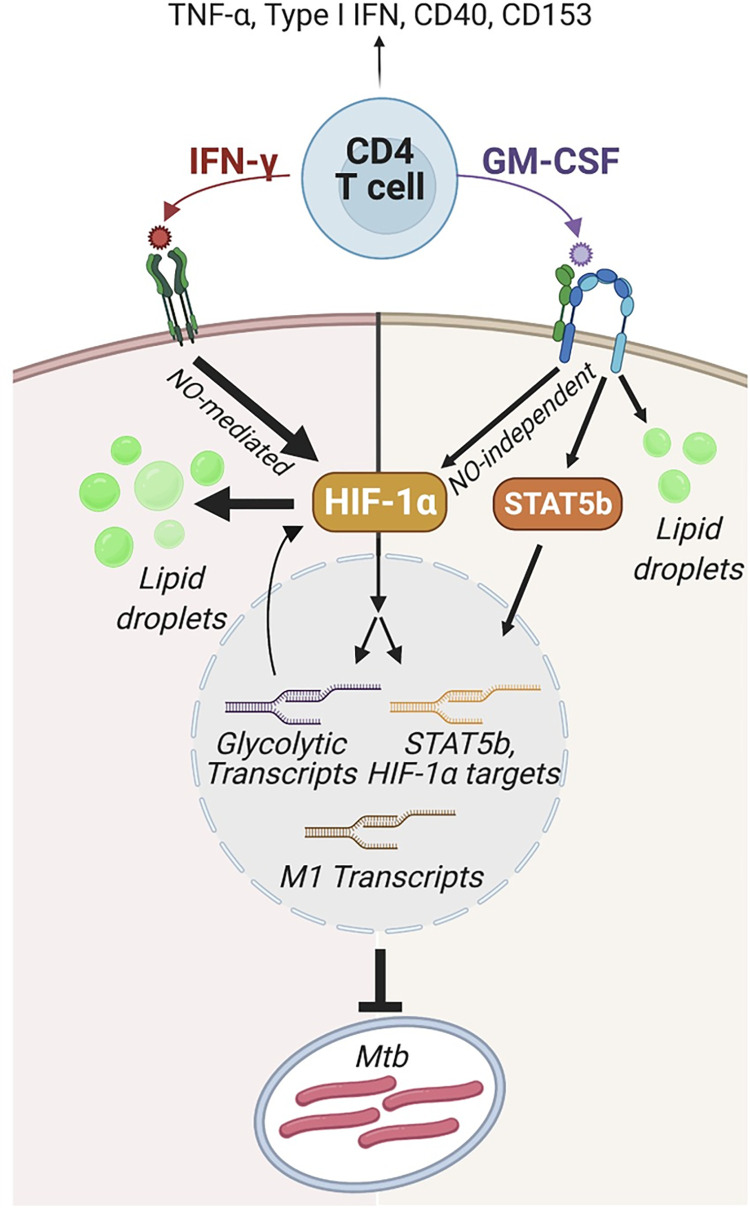
Model for IFN-γ-independent CD4 T cell-mediated control of *M*. *tuberculosis* by GM-CSF and HIF-1α. CD4 T cells secrete immune activating cytokines including TNF-α and Type I IFN and express the cell surface molecules CD40 and CD153, none of which mediate control of *M*. *tuberculosis* in macrophages. IFN-γ activates macrophages to control *M*. *tuberculosis* in part by stabilizing HIF-1α expression in a nitric oxide (NO)-dependent manner to drive a metabolic switch to glycolysis and the production of macrophage lipid droplets, and HIF-1α is required for IFN-γ-mediated control. In the absence of IFN-γ, CD4 T cell-derived GM-CSF is necessary for *M*. *tuberculosis* control in BMDMs and GM-CSF requires HIF-1α expression for control. HIF-1α activation during GM-CSF-mediated control, however, is NO-independent, indicating distinct mechanisms of activation following IFN-γ and GM-CSF signaling. Similarly, both IFN-γ and GM-CSF mediate lipid droplet biogenesis, but this is HIF-1α-dependent following IFN-γ activation and HIF-1α-independent in the context of GM-CSF. Importantly, lipid droplets do not mediate bacterial control following IFN-γ or GM-CSF activation. With or without IFN-γ, CD4 T cells producing GM-CSF upregulate macrophage expression of M1 and glycolytic transcripts leading to an activated, polarized, and antibacterial state which controls bacterial growth through an undefined mechanism. Model created using BioRender.com.

The antibacterial mechanisms downstream of GM-CSF signaling are still unclear, so we chose to focus on peritoneal macrophages where rGM-CSF is sufficient for bacterial control. Since IFN-γ-independent control of *M*. *tuberculosis* by lung CD4 T cells is NO-independent and requires HIF-1α (Figs 3B and 4I), we tested a role for NO and HIF-1α in GM-CSF-mediated control in peritoneal macrophages. Like CD4 T cell-mediated control, GM-CSF treatment of *M*. *tuberculosis*-infected macrophages treatment led to an increase in glucose uptake and an increase in the number and size of macrophage LDs (Figs 6D–6I and S5D)—both prominent effects of HIF-1α activation following IFN-γ signaling—and led to a moderate increase in HIF-1α protein levels independent of NO (Figs 6J and S5C). GM-CSF induced LDs were not required for control (S5E and S5F Fig), as expected [27,31]. Like *Hif1a*^*-/-*^ BMDMs, *Hif1a*^*-/-*^ peritoneal macrophages have a defect in control mediated by IFN-γ (Fig 6K) and, like CD4 T cell co-cultures, *Hif1a*^*-/-*^ peritoneal macrophages have a defect in control mediated by GM-CSF (Fig 6K). A requirement for HIF-1α in the absence of NO indicates that the mechanism of HIF-1α activation following GM-CSF signaling is distinct from the NO-mediated HIF-1α stabilization initiated by IFN-γ. Moreover, unlike IFN-γ-induced LDs, GM-CSF-mediated LD formation was HIF-1α-independent (Fig 6E–6I). Therefore, IFN-γ and GM-CSF not only stimulate distinct pathways of HIF-1α activation, but they also lead to distinct effects of HIF-1α activation, with HIF-1α mediating LD formation only after IFN-γ stimulation (Fig 7). As LD formation is uncoupled from control in both contexts, it is likely that the antibacterial effects of HIF-1α activation are related during IFN-γ- and GM-CSF-mediated control, although how HIF-1α activation leads to macrophage control of infection remains an open question.

## Discussion

Immunity to *M*. *tuberculosis* requires CD4 T cells and IFN-γ to activate effector functions within infected macrophages. This is supported by the extreme susceptibility of *Ifng*^*-/-*^ and *Rag1*^*-/-*^ mice, the high co-morbidity between TB and low CD4 T cell counts caused by AIDS, and the ability of recombinant IFN-γ to control bacterial growth in mouse and human macrophages *in vitro* [1–6,32]. However, while IFN-γ production by CD4 T cells is most likely necessary for full control of infection, it has become clear that IFN-γ is a poor biomarker for effective CD4 T cell immunity to TB and that CD4 T cells are capable of controlling infection even in the absence of IFN-γ [1,7,33]. Here, we use an *in vitro* CD4 T cell-macrophage co-culture model to confirm the finding that CD4 T cells can control *M*. *tuberculosis* in the absence of IFN-γ, and we demonstrate that this control is mediated by a secreted, antibacterial effector produced by lung-derived CD4 T cells and by *in vitro* differentiated Th1 and Th17.1 T cells.

RNA sequencing of *M*. *tuberculosis*-infected BMDMs after CD4 T cell co-culture revealed an IFN-γ-independent program of HIF-1α-driven transcription and significant upregulation of genes involved in glycolysis, reminiscent of the effects observed following IFN-γ activation. CD4 T cells also induced the development of macrophage LDs—a canonical effect of HIF-1α stabilization—in the absence of IFN-γ signaling. LDs support macrophage defense against infection, and lung granulomas in TB patients are surrounded by “foamy macrophages” that contain large accumulations of LDs [34,35]. Importantly, we demonstrate that HIF-1α expression is required for IFN-γ-independent control and show that, unlike HIF-1α stabilization following IFN-γ signaling, CD4 T cells stabilize HIF-1α in *Ifngr*^*-/-*^ macrophages in the absence of NO. These findings help clarify the role of NO during *M*. *tuberculosis* infection. While NO has long been thought to be directly bactericidal to *M*. *tuberculosis*, the regulatory role of NO as a second messenger has gained recent appreciation [36,37], including work from our laboratory showing that HIF-1α and iNOS form a positive feedback loop required for IFN-γ-mediated control of *M*. *tuberculosis* in BMDMs [26]. We have also shown that HIF-1α and iNOS participate in the same pathway following IFN-γ activation of BMDMs [26]. IFN-γ- and NO-independent HIF-1α stabilization during CD4 T cell co-culture positions NO upstream of HIF-1α following IFN-γ signaling and implicates NO as primarily a regulatory, rather than bactericidal, molecule during *M*. *tuberculosis* infection, at least in the context of CD4 T cell activation of macrophages. Furthermore, the need for HIF-1α expression to mediate both IFN-γ-mediated and IFN-γ-independent CD4 T cell control emphasizes the central role of HIF-1α in cell-intrinsic control of infection and indicates that these two pathways of macrophage activation converge on HIF-1α activation. Finally, we show that GM-CSF control of *M*. *tuberculosis* in peritoneal macrophages requires HIF-1α. This is the first demonstration of a functional link between GM-CSF and HIF-1α during *M*. *tuberculosis* infection, although this relationship has precedence in the literature in other contexts. In mouse neural progenitor cells GM-CSF treatment induces a PI3K–NF-κB signaling pathway that increases HIF-1α expression [38], and in a mouse melanoma model GM-CSF treatment induces macrophage secretion of vascular endothelial growth factor (VEGF) in a HIF-1α-dependent manner [39]. Research into the signaling pathways that mediate NO-dependent and -independent HIF-1α stabilization will help elucidate mechanisms of cell-intrinsic control of *M*. *tuberculosis*.

The ligand-receptor pair CD30 ligand (*Tnfsf8*), also known as CD153, and CD30 (*Tnfrsf8*) has generated excitement as a potentially important IFN-γ-independent signaling pathway during *M*. *tuberculosis* infection. In humans, higher frequencies of *M*. *tuberculosis* specific CD153+ CD4 T cells correlate with a lower lung bacterial load [40], and *Tnfsf8*^*-/-*^ mice have earlier mortality than wild-type following infection [28]. However, data point to a regulatory, rather than bactericidal, role for CD153, particularly in the lung. *Tnfsf8* expression is mostly dispensable for control of infection outside of the lungs, and the overabundance of IFN-γ-producing CD4 T cells observed in the lungs of *Tnfsf8*^*-/-*^ mice following infection has been shown to drive immunopathology [13,40]. In a similar manner, reconstitution of *Rag1*^*-/-*^ mice with CD4 T cells that overexpress IFN-γ induces pulmonary pathology and exacerbates bacterial burden in the lung while controlling infection outside of the lungs [13]. *Rag1*^*-/-*^ mice reconstituted with *Tnfsf8*^*-/-*^ CD4 T cells have decreased survival compared to wild-type, but lowering the frequency of IFN-γ-producing CD4 T cells in the lung by reconstitution with a 1:1 ratio of *Tnfsf8*^*-/-*^ and *Ifng*^*-/-*^ CD4 T cells rescues this phenotype [28]. We show that BMDMs have low expression of *Tnfrsf8* at baseline and after CD4 T cell co-culture, and that BMDMs deficient in *Ifngr* and *Tnfrsf8* have no loss of CD4 T cell-mediated control compared to macrophages lacking *Ifngr* alone. Collectively, these data point to a model where CD153 negatively regulates CD4 T cell IFN-γ production to control immune pathology, particularly in the lung, rather than signaling through CD30 on infected macrophages to induce cell-intrinsic control of bacterial replication.

Mouse and human T cells make GM-CSF during *M*. *tuberculosis* infection [15], and *Csf2*^*-/-*^ mice have a higher lung bacterial burden and succumb more quickly to disease than wild-type [16]. Chimeric mouse experiments with *Csf2*^*-/-*^ and wild-type mice show the GM-CSF production is particularly important for control of infection in the lung, with no effect in the spleen [15]. Furthermore, adoptive transfer of wild-type or *Csf2*^*-/-*^ CD4 T cells into wild-type or *Csf2*^*-/-*^ mice shows that CD4 T cell-derived GM-CSF is important *in vivo* only in the absence of non-hematopoietic GM-CSF [15]. These *in vivo* experiments are insufficient to conclude that CD4 T cell-derived GM-CSF acts directly on infected macrophages and are complicated by the immunoregulatory roles of GM-CSF, including the fact that *Csf2*^*-/-*^ mice and humans with deficiencies in GM-CSF Receptor signaling have a defect in alveolar macrophage development [41,42]. Our finding that GM-CSF is required for CD4 T cell-mediated IFN-γ-independent control is the first evidence that CD4 T cell-derived GM-CSF participates in cell-intrinsic control of *M*. *tuberculosis*.

While rGM-CSF is sufficient to restrict *M*. *tuberculosis* in peritoneal macrophages, it is necessary but not sufficient for control in BMDMs or alveolar macrophages and, surprisingly, does not rescue the lack of IFN-γ-independent control seen in co-cultures with *Csf2*^*-/-*^ CD4 T cells. One explanation for this discrepancy may be that *Csf2*^*-/-*^ CD4 T cells lack an additional effector produced by wild-type T cells that is required for GM-CSF-mediated control in BMDMs. This hypothesis is supported by literature showing that GM-CSF is important for the development, activation, and function of CD4 T cells [43]. *Csf2*^*-/-*^ mice have a diminished Th1 response compared to wild-type [44], which partially accounts for the increased susceptibility of *Csf2*^*-/-*^ mice to *M*. *tuberculosis* infection [16]. In anti-tumor immunity, GM-CSF treatment increases the frequency of tumor-specific Th1 T cells [45], and, in experimental autoimmune thyroiditis, GM-CSF treatment has been shown to both enhance IL-6–dependent Th17 cell responses and increase the number of immunoregulatory CD4+CD25+ T cells [46, 47]. Alternatively, there may be differences in the ability of CD4 T cell-derived and recombinant GM-CSF to effectively signal to BMDMs. *In vivo*, GM-CSF is subject to substantial post-translation modifications—primarily N- and O-glycosylation—with a molecular weight ranging from 14 to 32 kDa [48], and glycosylation of GM-CSF is required for effective signaling and superoxide induction in neutrophils [49]. Western blot analysis of the supernatant of *in vitro* differentiated Th1s revealed at least seven distinct species of GM-CSF, including a heavily glycosylated 32 kDa variant, compared to the single 14 kDa band for non-glycosylated recombinant GM-CSF (S5G Fig). Furthermore, the finding that HIF-1α is required for control in BMDMs by *Ifng*^*-/-*^ CD4 T cells and for control in peritoneal macrophages by rGM-CSF suggests similar mechanisms of action and argues that GM-CSF, rather than a second signal, from *Ifng*^*-/-*^ CD4 T cells is the critical factor that mediates IFN-γ-independent control. Further studies that delineate the role of GM-CSF in CD4 T cell development and macrophage activation will aid the development of vaccines capable of eliciting *M*. *tuberculosis*-specific GM-CSF-producing CD4 T cells and may expediate an end to the global TB pandemic.

## Methods

### Ethics statement

All procedures involving the use of mice were approved by the University of California, Berkeley Institutional Animal Care and Use Committee (protocol R353-1113B). All protocols conform to federal regulations and the National Research Council’s *Guide for the Care and Use of Laboratory Animals*.

### Reagents

Recombinant mouse IFN-γ was obtained from R&D Systems (485-MI) and used at 6.25 ng/mL. Recombinant mouse GM-CSF expressed in HEK293 cells was obtained from Sino Biological (51048-MNAH) and used at 10 ng/mL unless indicated. Proteinase K was obtained from Millipore Sigma (RPROTK-RO); 20 mg/mL stocks in ddH_2_O were diluted to a working concentration of 200 μg/mL in cell culture supernatant. 2-Deoxy-D-glucose (2-DG) was obtained from Sigma Aldrich (D8375) and used at 0.32 mM. Supernatant nitrite was measured by the Griess test as a proxy for NO production by mixing a solution of .1% napthylethylenediamine, 1% sulfanilamide, and 2% phosphoric acid 1:1 with supernatant and measuring absorbance at 546nm. Glucose uptake was determined using a glucose (HK) assay kit from Sigma-Aldrich (GAHK20) according to the manufacturer’s protocol.

### Mice

C57BL/6 (wild-type, strain 000664), B6.129S7-*Ifng*^*tm1Ts*^/J (*Ifng*^*-/-*^, strain 002287), B6.129P2-*Nos2*^*tm1Lau*^/J (*Nos2*^*-/-*^, strain 002609), and B6.129S-*Csf2*^*tm1Mlg*^/J (*Csf2*^*-/-*^, strain 026812) were purchased from The Jackson Laboratory and bred in-house. B6.129-*Hif1a*^*tm3Rsjo*^/J (*Hif1a*^*fl/fl*^, strain 007561) and B6.129P2-*Lyz2*^*tm1(cre)Ifo*^/J (LysMcre, strain 004781) mice were obtained from The Jackson Laboratory, crossed to generate *Hif1a*^*fl/fl*^, LysMcre^+/+^ (referred to here as *Hif1a*^*-/-*^) mice and bred in-house. *Ifngr1*^*-/-*^ (*Ifngr*^*-/-*^) mice were provided by D. Raulet. *Tnfrsf1a*^*-/-*^*/Tnfrsf1b*^*-/-*^ (*Tnfr1/2*^*-/-*^) mice were provided by G. Barton [50]. *Ifnar*^*-/-*^*/Ifngr1*^*-/-*^ (*Ifnar*^*-/-*^*/Ifngr*^*-/-*^) mice were provided by M. Welch [51]. *Csf2rb-/-* mice were provided by S. Shin [52]. C7 TCR tg.CD90.1 (C7 Tg) mice which express a T cell receptor specific for the *M*. *tuberculosis* antigen ESAT-6 were provided by K. Urdahl and have been described previously [53,54]. H11^Cas9^ CRISPR/Cas9 knock-in (Cas9 Tg) mice, which constitutively express CRISPR associated protein 9 (Cas9), were provided by R. Vance and were bred to *Ifngr*^*-/-*^ mice to generate *Ifngr*^*-/-*^*/*Cas9 Tg mice.

### Cell culture

Bone-marrow was obtained from wild-type, *Nos2*^*-/-*^, *Hif1a*^*-/-*^, *Ifngr*^*-/-*^, *Tnfr1/2*^*-/-*^, *Ifnar*^*-/-*^*/Ifngr*^*-/-*^, and *Csf2rb*^*-/-*^ mice and cultured in DMEM with 10% FBS, 2 mM L-glutamine, and 10% supernatant from 3T3–M-CSF cells (BMDM media) for 6 d with feeding on day 3 to generate bone marrow-derived macrophages (BMDMs). Peritoneal macrophages were obtained from wild-type and *Hif1a*^*-/-*^ mice by 5 mL ice-cold PBS lavage and cultured in RPMI with 10% FBS, 2 mM L-glutamine and 35 μg/mL kanamycin (peritoneal macrophage media) for 24 h to allow for macrophage adherence. Alveolar macrophages were isolated from wild-type mice by ten 1 mL lavages per mouse using PBS with 10% FBS and 2mM EDTA warmed to 37°C and an 18-G catheter. Cells were treated with ACK lysis buffer for 2 minutes at room temperature and cultured in RPMI with 10% FBS, 2mM L-glutamine, 1 mM sodium pyruvate and 50 μg/mL kanamycin (AM media).

### CRISPR/Cas9 gene targeting

Guide sequences targeting *Tnfrsf8* were selected from the murine Brie guide library and cloned into the pLentiGuidePuro backbone from Addgene (52963). Bone marrow was obtained from *Ifngr*^*-/-*^*/*Cas9 Tg mice, treated with ACK lysis buffer, and cultured in BMDM media. Lenti-X 293T cells from Takara Bio (632180) were co-transfected with psPAX2, pMD2.G, and pLentiGuidePuro containing target guide sequences using Lipofectamine 2000 from Invitrogen (11668019) according to the manufacturer’s protocol. The next day, 293T media was replaced with BMDM media for 24 hours to collect lentivirus, and *Ifngr*^*-/-*^/Cas9 Tg BMDMs were then transduced using 293T supernatant. On day 5, 4 μg/mL puromycin was added to select for a polyclonal population of cells. On day 12, *Ifngr*^*-/-*^*/Tnfrsf8*^*-/-*^ double-knockout BMDMs were harvested and infected with *M*. *tuberculosis* as described above. Targeting efficiency was determined by sequencing targeted genomic sites and analyzing population level genome editing using TIDE analysis (https://tide.nki.nl) [55]. *Tnfrsf8* was targeted in two independent experiments, each with three targeting guides. Data shown in Fig 4 are representative of results with gRNA 5’-AGACCTCAGCCACTACCCAG-3’ which had a genome editing efficacy of 91.3–91.9%.

### Bacterial cultures

Frozen stocks of low passage *M*. *tuberculosis* Erdman were grown to log phase over 5 d at 37°C in Middlebrook 7H9 media with 10% albumin-dextrose-saline, 0.5% glycerol, and 0.05% Tween-80. *M*. *tuberculosis* Erdman-mCherry was generated by D. Kotov and R. Vance by transforming wild-type Erdman with pMSP12::mCherry, a gift from Lalita Ramakrishnan (Addgene plasmid # 30169; http://n2t.net/addgene:30169;RRID:Addgene_30169) and was cultured as described above.

### *In vitro* infections

BMDMs were plated in 24-well or 96-well plates at 3 x 10^5^ or 5 x 10^4^ cells/well, respectively, allowed to adhere for 48 hours, and infected in DMEM with 5% horse serum and 5% FBS by 4-hour phagocytosis or 10 min spinfection at 300 rcf. Unless otherwise indicated, BMDMs were infected at a multiplicity of infection of 1. Peritoneal macrophages were plated in 12-well, 24-well or 96-well plates at 1.65 x 10^6^, 1.1 x 10^6^ or 1.5 x 10^5^ cells/well, respectively, cultured for 24 hours to allow for macrophage adherence, washed with PBS, and infected in peritoneal macrophage media with 5% horse serum by 10 min spinfection at 300 rcf. After infection, cells were washed once with PBS before replacing with the appropriate media. Alveolar macrophages were plated in 96-well plates at 5 x 10^4^ cells/well, allowed to adhere for 24 hours, and infected in DMEM with 5% horse serum and 5% FBS by 4-hour phagocytosis at a multiplicity of infection of 1. After infection, cells were washed once with PBS before replacing with the appropriate media. CFU/well was determined at the indicated time points by washing infected cells with PBS, lysing in sterile-filtered distilled water with 0.5% Triton-X100 for 10 min at 37°C, diluting in PBS prepared with 0.05% Tween 80, plating onto 7H10 plates supplemented with 0.5% glycerol and 10% OADC (Middlebrook), and incubating for 21 d at 37°C. CFU is reported as fold-change over inoculum (calculated by plating t = 0 CFU immediately after phagocytosis or spinfection), or as CFU/well, referring to the total number of bacteria per well of the assay plate. Luminescence emission of *M*. *tuberculosis* Erdman-lux was measured at 470 nm with a Spectra-L plate reader (Molecular Devices, San Jose, CA) and is reported as Relative Light Units (RLU).

### Lung-derived CD4 T cell co-cultures

CD4+ cells were isolated from the lungs of 7–12-week-old wild-type, *Ifng*^*-/-*^, *Nos2*^*-/-*^ or *Csf2*^*-/-*^ mice 21–28 d after ~500 CFU aerosol infection with *M*. *tuberculosis* Erdman using mouse CD4 (L3T4) MicroBeads from Miltenyi Biotec (130-117-043) according to the manufacturer’s protocol, resuspended in RPMI with 10% FBS, 2 mM L-glutamine, 1 mM Sodium pyruvate, 1 mM NEAA, 20 mM HEPES, 55 μM 2-mercaptoethanol and 35 μg/mL kanamycin (T cell media) supplemented with 10% supernatant from 3T3–M-CSF cells and added to infected BMDMs in co-culture or in transwells. Unless indicated, CD4 T cells were added at a 5:1 T cell:BMDM ratio. In these experiments, T cell media supplemented with 10% supernatant from 3T3–M-CSF cells was used for untreated and IFN-γ-treated controls.

### *In vitro* T cell differentiation

CD4+ cells were isolated from C7 Tg mouse spleens using mouse CD4 (L3T4) MicroBeads from Miltenyi Biotec (130-117-043) according to the manufacturer’s protocol and cultured in T cell media at 2 x 10^6^ cells/mL with 1 μM ESAT-6 peptide pool (NIH BEI Resources Repository catalog no. NR-34824) and 2 x 10^6^ cells/mL irradiated wild-type splenocytes (3200 rads). To differentiate Th1s, 10 U/mL IL-2 from R&D Systems (1150-ML), 5 U/mL IL-12 p70 from PeproTech (210–12), and 1 μg/mL rat anti-mouse IL-4 from R&D Systems (MAB404100) was added; for Th17.1s, 2 ng/mL TGF-β from Invitrogen (14834262), 20 ng/mL IL-6 from PeproTech (216–16), 2.5 μg/mL rat anti-mouse IFN-γ from Biolegend (505801) and 1 μg/mL anti-IL-4 was added; and for Th2s, 10 U/mL IL-2, 10 ng/mL IL-4 from R&D Systems (404-ML), and 3 μg/mL anti-IFN-γ was added. On day 3, cells were collected and replated at 2 x 10^6^ cells/mL with 10 U/mL IL-2 (Th1s), 2 ng/mL TGF-β, 10 ng/mL IL-6, 30 ng/mL IL-1β from R&D Systems (401-ML), 0.5 μg/mL anti-IFN-γ, and 0.33 μg/mL anti-IL-4 (Th17.1s), or 10 U/mL IL-2, 10 ng/mL IL-4, and 1 μg/mL anti-IFN-γ (Th2s). Differentiated T cells were harvested on day 5 and used in co-culture at a 5:1 T cell:BMDM ratio or were replated at 2 x 10^6^ cells/mL in T cell media and stimulated overnight with 100 U/mL IL-2 (Th1s), 5 ng/mL IL-1β and 5 ng/mL IL-23 from Invitrogen (14823163) (Th17.1s) or 100 U/mL IL-2 and 100 ng/mL IL-4 (Th2s) in plates coated with 10 μg/mL Armenian hamster anti-mouse CD3Ɛ (BE0001-1) and 2 μg/mL Syrian hamster anti-mouse CD28 (BE0015-1) from Bio X Cell to generate Th1, Th17.1, or Th2 supernatants. On day 6, T cell supernatants were collected and supplemented with 10% supernatant from 3T3–M-CSF cells before addition to BMDMs. In these experiments, T cell media supplemented with 10% supernatant from 3T3–M-CSF cells was used for untreated and IFN-γ-treated controls.

### Cytokine profiling

Differentiated Th1 cells were harvested on day 5, replated at 2 x 10^6^ cells/mL in Opti-MEM media (Gibco) plus 2 mM L-glutamine, 1 mM Sodium pyruvate, 1 mM NEAA, 20 mM HEPES and 55 μM 2-mercaptoethanol (Opti-MEM T cell media) and stimulated overnight as described above. On day 6, T cell supernatants were collected, and cytokine profiling was performed by the Stanford Human Immune Monitoring Center (Stanford, CA) using a mouse 48-plex immunoassay (Procarta).

### RNA sequencing

For RNA-seq on BMDMs after lung CD4 T cell co-culture (Figs 2 and S2), four independent experiments were performed. For each experiment, BMDMs were seeded in 24-well plates, infected as described, and co-cultured with lung CD4 T cells isolated as described. At 24 hours postinfection, CD4 T cells were separated from BMDMs using CD4 (L3T4) Dynabeads from Invitrogen (11445D) according to the manufacturer’s protocol, and BMDMs were lysed in 1 mL TRIzol (Invitrogen). Total RNA was extracted using chloroform and 2 mL Heavy Phase Lock Gel tubes from QuantaBio (2302830), and the aqueous layer was further purified using RNeasy Mini spin columns from Qiagen (74104). RNA quality was determined using an Agilent 2100 Bioanalyzer and RNA concentration was determined using the Qubit Quantitation Platform at the Functional Genomics Laboratory of The California Institute for Quantitative Biosciences (University of California, Berkeley). RNA-seq libraries were prepared by the DNA Technologies and Expression Analysis Core (University of California, Davis) and differential gene expression was analyzed by the Genome Center and Bioinformatics Core Facility (University of California, Davis). Data displayed as “RNA-seq reads” were normalized to counts per million.

### HIF-1α western blot

BMDMs infected for 12 hours at a multiplicity of infection of 5 as described or peritoneal macrophages infected for 20 hours at a multiplicity of infection of 5 as described were washed with ice cold PBS, lysed in 1X SDS-PAGE buffer on ice and heat sterilized for 20 min at 100°C for removal from the BSL3 facility. Total protein lysate was analyzed by SDS-PAGE using precast 4–20% Criterion TGX protein gels from Bio-Rad Laboratories (5671093), rabbit mAb to HIF-1α (D2U3T) from Cell Signaling Technology (14179S), and an HRP-conjugated secondary antibody. Blots were developed with Western Lightning Plus-ECL chemiluminescence substrate from PerkinElmer (NEL105001EA) and a ChemiDoc MP system from Bio-Rad Laboratories.

### GM-CSF ELISA

GM-CSF concentration in cell culture supernatant was determined using the Mouse GM-CSF Quantikine ELISA kit from R&D Systems (MGM00) according to the manufacturer’s protocol.

### Microscopy

For microscopy to detect lipid droplets, BMDMs or peritoneal macrophages were plated at 5 x 10^4^ or 1.5 x 10^5^ cells/well, respectively, in black, clear bottom 96-well plates and infected with *M*. *tuberculosis* Erdman-mCherry as described above at a multiplicity of infection of 4 (BMDMs) or 2 (peritoneal macrophages). At the indicated time points, cells were fixed in 4% paraformaldehyde, washed with PBS and stained for neutral lipids with BODIPY 493/503 from Invitrogen (D3922) at 1 μg/mL and with DAPI. All imaging was done on a Perkin Elmer Opera Phenix Automated Microscopy System and images were analyzed and quantified using CellProfiler.

### Statistical Analysis

Analysis of statistical significance was performed using GraphPad Prism 8 (GraphPad, La Jolla, CA).

### Dryad DOI

https://doi.org/10.5061/dryad.s7h44j18x [56].

## Supporting information

S1 Text Supporting Information title page, Extended Methods, and References. (DOCX)

S1 Fig Related to Fig 1, Lung-derived and in vitro-differentiated CD4 T cells control M. tuberculosis growth independent of IFN-γ. (**A**)-(**B**) CFU/well (A) or RLU fold-change (B) at d 4 postinfection for wild-type (A) or *Ifngr*^*-/-*^ BMDMs (B) co-cultured with the indicated ratios of lung-derived wild-type CD4 T cells to BMDMs. (**C**) Representative flow cytometry plots showing gating strategy and log10 fluorescence and percentage of Th1 or Th17.1 T cells that produce IFN-γ or IL-17 or express RORγt. (**D**) RLU fold-change at d 4 postinfection for *Ifngr*^*-/-*^ BMDMs treated with neat or diluted Th1 supernatants (sups). (**E**) Nuclei count per field of view at 24 h postinfection for wild-type and *Ifngr*^*-/-*^ BMDMs treated with Th1 sups. Figures are representative of two (A), (D) or at least three (B)-(C), (E) independent experiments. Error bars are SD from four replicate samples (A)-(B), (D) or 48 images from 4 replicate wells (E), **p<0.01, ***p<0.001, ****p<0.0001 by unpaired t-test; p-values in (B) are relative to UT.
(TIF)

S2 Fig Related to Fig 2, IFN-γ is not required for T cell-mediated macrophage polarization and activation during M. tuberculosis infection. (**A**) GSEA enrichment plots from the MSigDB C2 Curated gene sets for *Ifngr*^*-/-*^ BMDMs co-cultured with lung-derived wild-type CD4 T cells at d 1 postinfection. (**B**) Cytoscape enrichment map visualization of GSEA using the MSigDB C2 Curated gene sets comparing wild-type or *Ifngr*^*-/-*^ BMDMs co-cultured with lung-derived wild-type CD4 T cells or wild-type BMDMs + IFN-γ to untreated for each genotype at d1 postinfection. Positive phenotype = CD4 co-cultured or IFN-γ treated; negative phenotype = untreated. Gene set size is indicated by the size of node and similarity coefficient is indicated by edge width. Figures represent data from four independent experiments.
(TIF)

S3 Fig Related to Fig 3, CD4 T cells induce IFN-γ- and NO-independent increases in glycolytic gene expression, and to Fig 4, IFN-γ-independent control requires the transcription factor HIF-1α. (**A**) Glucose consumption at 48 h postinfection for wild-type and *Ifngr*^*-/-*^ BMDMs co-cultured with lung-derived wild-type CD4 T cells. (**B**) RLU fold-change at d 6 postinfection for wild-type, *Ifngr*^*-/-*^ and *Nos2*^*-/-*^ BMDMs co-cultured with a 10:1 ratio of lung-derived wild-type or *Nos2*^*-/-*^ CD4 T cells. (**C**) oPOSSUM bioinformatic prediction of transcription factors responsible for regulation of all genes found by RNA sequencing to be upregulated in *Ifngr*^*-/-*^ BMDMs after co-culture with lung-derived CD4 T cells compared to UT. (**D**) RLU fold-change at d 4 postinfection for wild-type and *Ifngr*^*-/-*^ BMDMs treated with Th1 supernatants (sups) and 2-DG. Figures represent data from three independent experiments (C) or are representative of two independent experiments (A)-(B), (D). Error bars are SD from four replicate samples, *p<0.05, **p<0.01, ****p<0.0001 by unpaired t-test.
(TIF)

S4 Fig Related to Fig 5, IFN-γ-independent control of M. tuberculosis requires CD4 T cell-derived GM-CSF. (**A**) CFU fold-change at d 5 postinfection for *Ifngr*^*-/-*^ BMDMs co-cultured with lung-derived wild-type CD4 T cells and treated with anti-CD40. (**B**) RNA-seq reads of *Tnfrsf8* at 24 h postinfection in wild-type and *Ifngr*^*-/-*^ BMDMs co-cultured with lung-derived CD4 T cells. (**C**)-(**D**) ELISA for GM-CSF concentration in (C) *in vitro* differentiated Th1 and Th17.1 T cell supernatants (sups) or (D) lung-derived CD4 co-culture sups at d 2 postinfection. (**E**)-(**F**) RNA-seq reads of (E) *Csf2ra* and (F) *Csf2rb* at 24 h postinfection in wild-type and *Ifngr*^*-/-*^ BMDMs co-cultured with lung-derived CD4 T cells. (**G**) GSEA enrichment plot for STAT5B_01 from the MSigDB C3: Curated Transcription Factor Target Prediction gene sets for *Ifngr*^*-/-*^ BMDMs co-cultured with lung-derived CD4 T cells at d 1 postinfection. Figures represent data from four independent experiments (B), (E)-(G) or are representative of three independent experiments (A), (C)-(D). Error bars are SD from four independent experiments (B), (E)-(F) or three (C) or four (A), (D) replicate samples, *p<0.05, ***p<0.001, ****p<0.0001 by unpaired t-test.
(TIF)

S5 Fig Related to Fig 6, GM-CSF activates HIF-1α to restrict M. tuberculosis in peritoneal macrophages. (**A**) CFU fold-change at d 4 postinfection for BMDMs and peritoneal macrophages treated with GM-CSF from the indicated sources. (**B**) CFU fold-change at d 5 postinfection for *Ifngr*^*-/-*^ BMDMs co-cultured with wild-type or *Csf2*^*-/-*^ lung-derived CD4 T cells and treated with GM-CSF from the indicated sources. (**C**) Griess assay at 24 h postinfection for BMDMs and peritoneal macrophages treated with GM-CSF. (**D**) Quantification of Fig 6E–6H for % lipid droplet (LD)-positive macrophages. (**E**)-(**F**) Quantification of (E) LD signal / nuclei at d 1 postinfection and (F) bacteria count / nuclei at d 2 postinfection for peritoneal macrophages treated with 1 ng/mL GM-CSF and the DGAT1 inhibitor T863. (**G**) Western blot for GM-CSF comparing a dose response of recombinant GM-CSF to three biological replicates of 1 mL TCA-precipitated C7 Th1 supernatants (sups). Figures are representative of two (B)-(F) or at least three (A), (G) independent experiments. Error bars are SD from four replicate samples (A)-(C) or four replicate wells (D)-(F), *p<0.05, **p<0.01, ***p<0.001, ****p<0.0001 by unpaired t-test; p-values in (B) are relative to UT.
(TIF)

S1 Table Genes with differential expression greater than 2-fold compared to untreated in both wild-type and *Ifngr*^*-/-*^ BMDMs during CD4 T cell co-culture. (XLSX)
