# Supplementary material for: IFN-γ-independent control of M. tuberculosis requires CD4 T cell-derived GM-CSF and activation of HIF-1α
Source: PLoS Pathog. 2022 Jul 25;18(7):e1010721. doi: 10.1371/journal.ppat.1010721 (PMC9352196; doi:10.1371/journal.ppat.1010721)
Supplement: S1 Text — (DOCX) [file ppat.1010721.s001.docx]

**Supplemental Information: Extended Methods, References, and Figures S1 – S5.**

**IFN-γ-independent control of *M. tuberculosis* requires CD4 T cell-derived GM-CSF and activation of HIF-1α**

Erik Van Dis^a,+^, Douglas M Fox^a^, Huntly M Morrison^a^, Daniel M Fines^a^, Janet Peace Babirye^a^, Lily H McCann^b^, Sagar Rawal^a^, Jeffery S Cox^a^, and Sarah A Stanley^a,b,*^

^a^Department of Molecular and Cell Biology, Division of Immunology and Pathogenesis, University of California, Berkeley, Berkeley, California, United States of America

^b^School of Public Health, Division of Infectious Diseases and Vaccinology, University of California, Berkeley, Berkeley, California, United States of America

^+^Current address: Departments of Immunology and Medicine, University of Washington, Seattle, Washington, United States of America

^*^sastanley@berkeley.edu

Page S2: Extended Methods, References

Page S3: Figure S1

Page S4: Figure S2

Page S5: Figure S3

Page S6: Figure S4

Page S7: Figure S5

**Extended Methods**

**Reagents**

GM-CSF expressed in *E. coli* was obtained from R&D Systems (415-ML) and GM-CSF expressed in Chinese hamster ovary cells was obtained from MedChemExpress (HY-P7069) and used at 10 ng/mL. Armenian hamster anti-mouse CD40 (102907) was obtained from Biolegend and used at 20 μg/mL. The DGAT1 inhibitor T863 was obtained from Sigma-Aldrich (SML0539) and used at 2.5 μM.

**Flow cytometry**

Th1 and Th17.1 T cells were generated as described and harvested on day 5. Cells were incubated with 1X Protein Transport Inhibitor Cocktail from eBioscience (00-4980-03) for 5 hrs at 37˚C and 5% CO_2_, incubated in 1:250 Fc block from Biolegend (101320) for 15 min at 4˚C, stained with anti-CD4 BUV737 from BD Biosciences (564933) and anti-MHC II FITC from Biolegend (107606) for 30 min at 4˚C, fixed and permeabilized for 30 min at room temperature (RT), and stained with anti-IFN-γ A647 from Biolegend (505816), anti-IL-17 BUV395 from BD Biosciences (565246), and anti-RORγt PerCP-e710 from eBioscience (46-6981-82) for 30 min at RT. Data were collected using a BD LSR Fortessa flow cytometer with FACSDiva software (BD Biosciences) and analyzed using FlowJo Software (Tree Star, Ashland, OR).

**Gene Set Enrichment Analysis network visualization**

Enrichment mapping was accomplished using Cytoscape and 1000 permutations of GSEA results from the MsigDB C2 Curated gene sets generated as described. Gene sets larger than 500 or smaller than 15 and clusters with < 3 gene sets were excluded from visualization. Only gene sets with p-value < 0.001 and FDR q-value < 0.01 were included and edges were displayed if Jaccard Overlap Combined > 0.375.

**Transcription factor prediction**

oPOSSUM has been described previously (1) and was used to predict macrophage transcription factors regulated by CD4 T cells during IFN-γ independent control of infection. oPOSSUM analysis was run on all genes in the RNAseq data found to be expressed at a higher level in *Ifngr^-/-^* BMDMs after co-culture with lung-derived CD4 T cells compared to UT.

**GM-CSF western blot**

C7 Th1 supernatants were generated in OptiMEM T cell media as described. Protein from 1 mL supernatant was concentrated by TCA precipitation and compared to a dose response of recombinant GM-CSF by SDS-PAGE using precast 4-20% Criterion TGX protein gels from Bio-Rad Laboratories (5671093), rabbit polyclonal Ab to mouse GM-CSF from Abcam (ab9741), and an HRP-conjugated secondary antibody. Blots were developed as described in the main text.

**References**

1. Ho Sui SJ, Mortimer JR, Arenillas DJ, Brumm J, Walsh CJ, Kennedy BP, Wasserman WW. 2005. oPOSSUM: identification of over-represented transcription factor binding sites in co-expressed genes. Nucleic Acids Research 33:3154-3164.
