# Supplementary figures and images for: IFN-γ-independent control of M. tuberculosis requires CD4 T cell-derived GM-CSF and activation of HIF-1α
Source: PLoS Pathog. 2022 Jul 25;18(7):e1010721. doi: 10.1371/journal.ppat.1010721 (PMC9352196; doi:10.1371/journal.ppat.1010721)

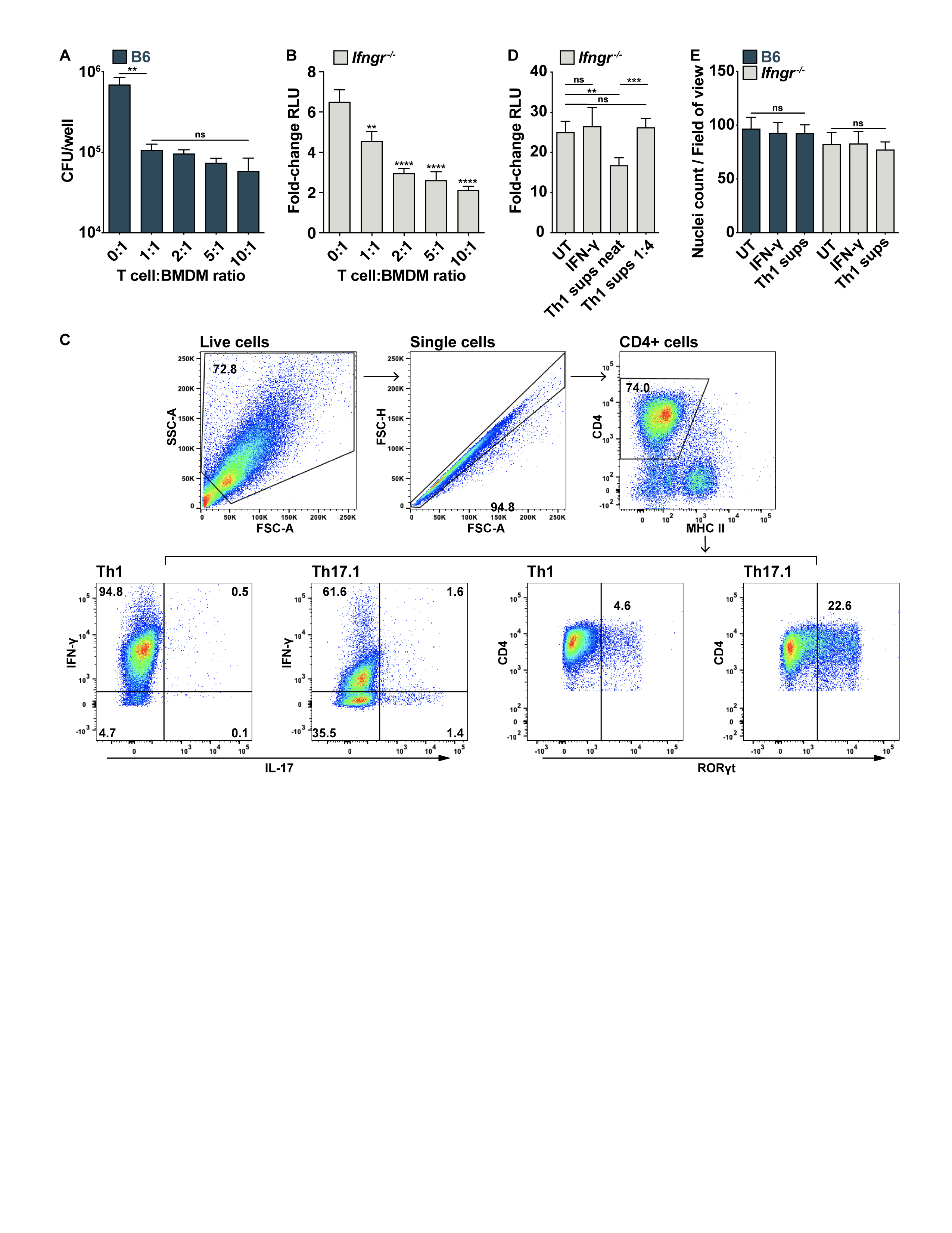

Supplement: S1 Fig — (A)-(B) CFU/well (A) or RLU fold-change (B) at d 4 postinfection for wild-type (A) or Ifngr-/- BMDMs (B) co-cultured with the indicated ratios of lung-derived wild-type CD4 T cells to BMDMs. (C) Representative flow cytometry plots showing gating strategy and log10 fluorescence and percentage of Th1 or Th17.1 T cells that produce IFN-γ or IL-17 or express RORγt. (D) RLU fold-change at d 4 postinfection for Ifngr-/- BMDMs treated with neat or diluted Th1 supernatants (sups). (E) Nuclei count per field of view at 24 h postinfection for wild-type and Ifngr-/- BMDMs treated with Th1 sups. Figures are representative of two (A), (D) or at least three (B)-(C), (E) independent experiments. Error bars are SD from four replicate samples (A)-(B), (D) or 48 images from 4 replicate wells (E), **p<0.01, ***p<0.001, ****p<0.0001 by unpaired t-test; p-values in (B) are relative to UT. (TIF) [file ppat.1010721.s002.tif]

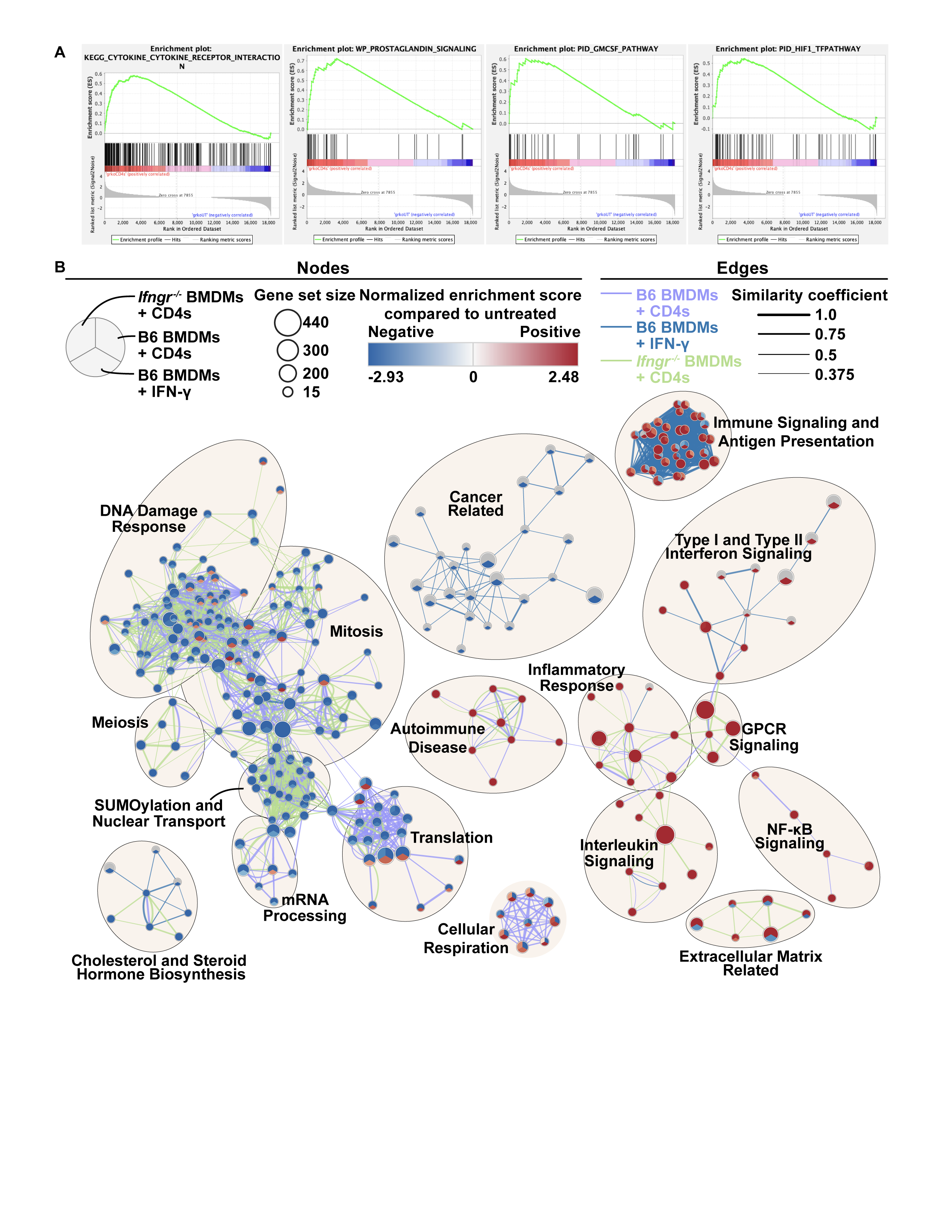

Supplement: S2 Fig — (A) GSEA enrichment plots from the MSigDB C2 Curated gene sets for Ifngr-/- BMDMs co-cultured with lung-derived wild-type CD4 T cells at d 1 postinfection. (B) Cytoscape enrichment map visualization of GSEA using the MSigDB C2 Curated gene sets comparing wild-type or Ifngr-/- BMDMs co-cultured with lung-derived wild-type CD4 T cells or wild-type BMDMs + IFN-γ to untreated for each genotype at d1 postinfection. Positive phenotype = CD4 co-cultured or IFN-γ treated; negative phenotype = untreated. Gene set size is indicated by the size of node and similarity coefficient is indicated by edge width. Figures represent data from four independent experiments. (TIF) [file ppat.1010721.s003.tif]

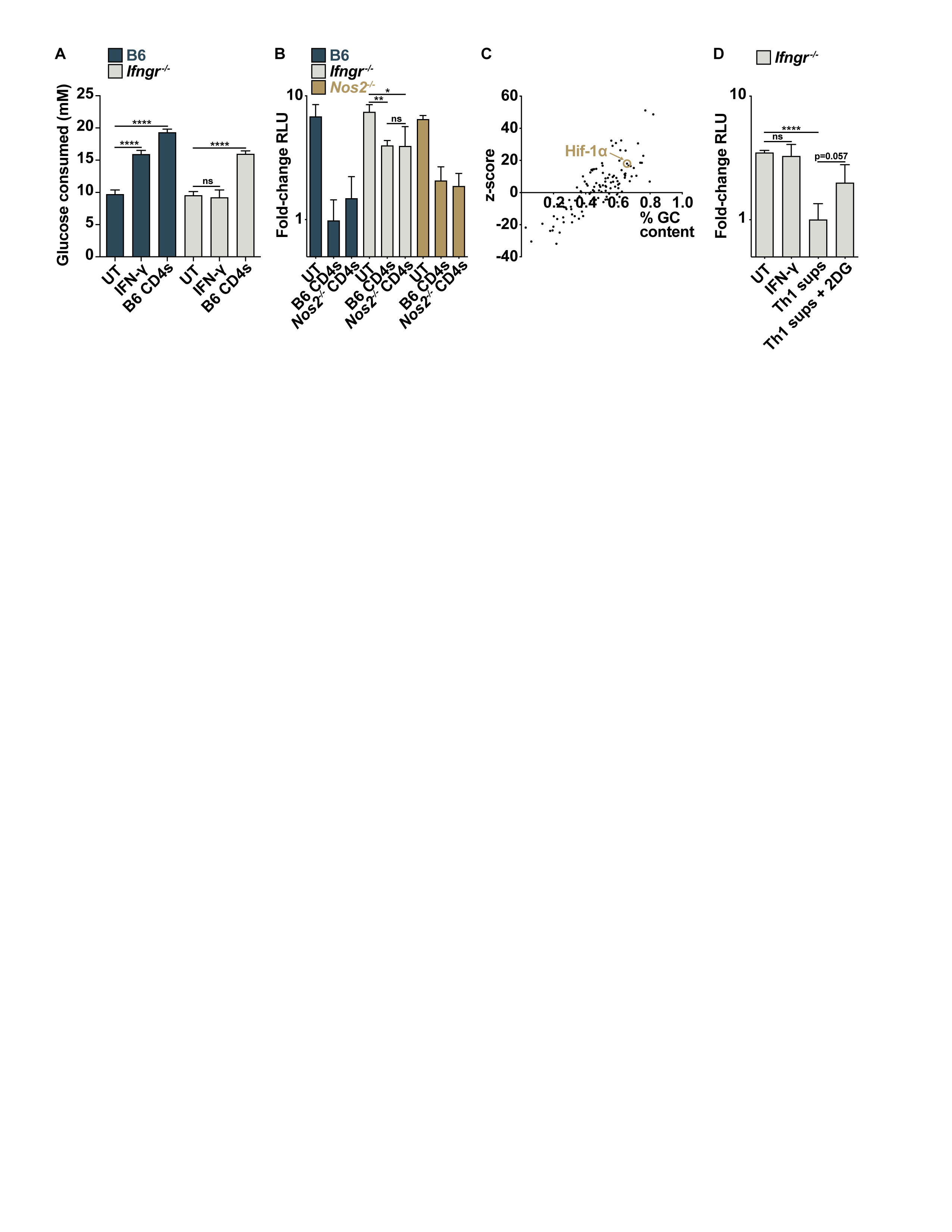

Supplement: S3 Fig — (A) Glucose consumption at 48 h postinfection for wild-type and Ifngr-/- BMDMs co-cultured with lung-derived wild-type CD4 T cells. (B) RLU fold-change at d 6 postinfection for wild-type, Ifngr-/- and Nos2-/- BMDMs co-cultured with a 10:1 ratio of lung-derived wild-type or Nos2-/- CD4 T cells. (C) oPOSSUM bioinformatic prediction of transcription factors responsible for regulation of all genes found by RNA sequencing to be upregulated in Ifngr-/- BMDMs after co-culture with lung-derived CD4 T cells compared to UT. (D) RLU fold-change at d 4 postinfection for wild-type and Ifngr-/- BMDMs treated with Th1 supernatants (sups) and 2-DG. Figures represent data from three independent experiments (C) or are representative of two independent experiments (A)-(B), (D). Error bars are SD from four replicate samples, *p<0.05, **p<0.01, ****p<0.0001 by unpaired t-test. (TIF) [file ppat.1010721.s004.tif]

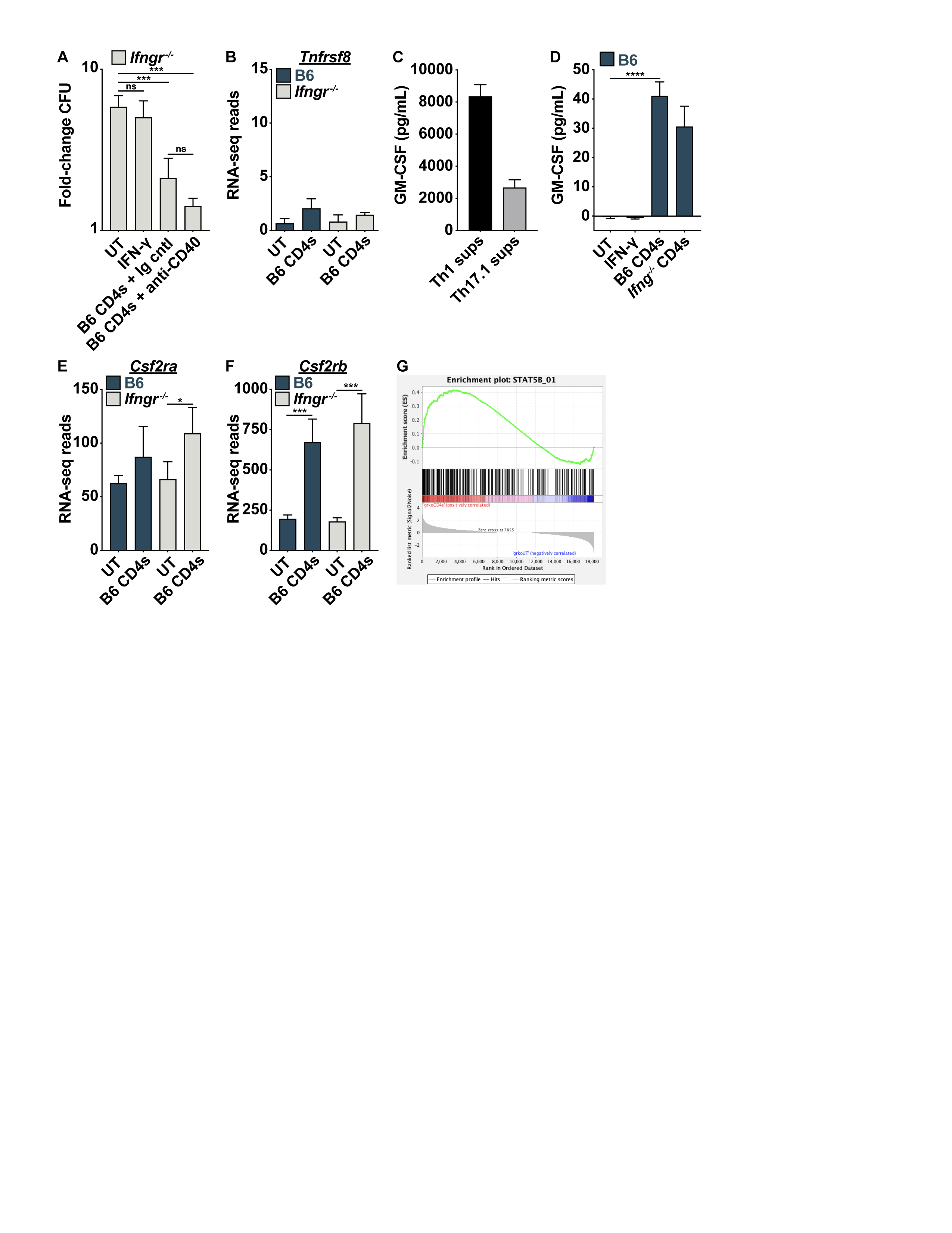

Supplement: S4 Fig — (A) CFU fold-change at d 5 postinfection for Ifngr-/- BMDMs co-cultured with lung-derived wild-type CD4 T cells and treated with anti-CD40. (B) RNA-seq reads of Tnfrsf8 at 24 h postinfection in wild-type and Ifngr-/- BMDMs co-cultured with lung-derived CD4 T cells. (C)-(D) ELISA for GM-CSF concentration in (C) in vitro differentiated Th1 and Th17.1 T cell supernatants (sups) or (D) lung-derived CD4 co-culture sups at d 2 postinfection. (E)-(F) RNA-seq reads of (E) Csf2ra and (F) Csf2rb at 24 h postinfection in wild-type and Ifngr-/- BMDMs co-cultured with lung-derived CD4 T cells. (G) GSEA enrichment plot for STAT5B_01 from the MSigDB C3: Curated Transcription Factor Target Prediction gene sets for Ifngr-/- BMDMs co-cultured with lung-derived CD4 T cells at d 1 postinfection. Figures represent data from four independent experiments (B), (E)-(G) or are representative of three independent experiments (A), (C)-(D). Error bars are SD from four independent experiments (B), (E)-(F) or three (C) or four (A), (D) replicate samples, *p<0.05, ***p<0.001, ****p<0.0001 by unpaired t-test. (TIF) [file ppat.1010721.s005.tif]

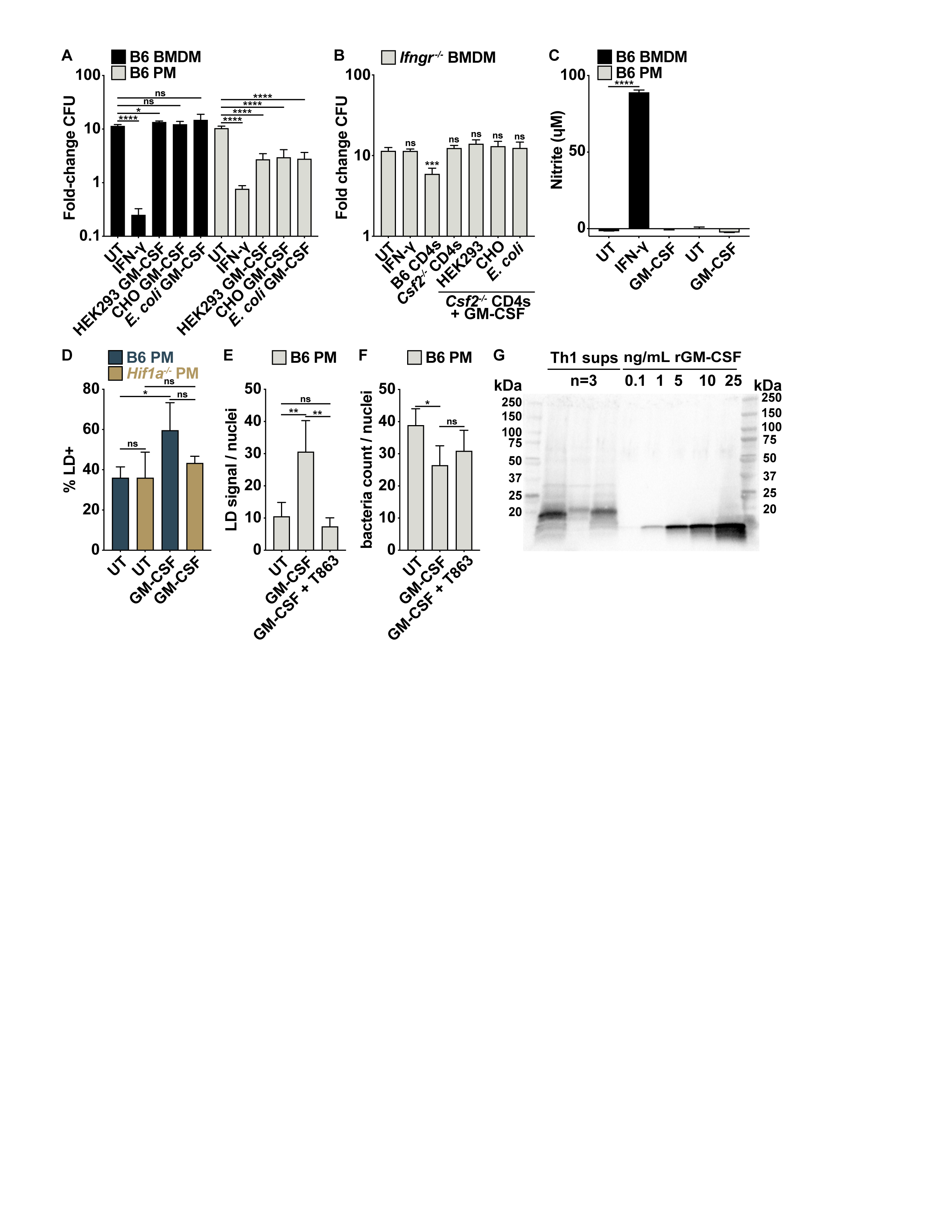

Supplement: S5 Fig — (A) CFU fold-change at d 4 postinfection for BMDMs and peritoneal macrophages treated with GM-CSF from the indicated sources. (B) CFU fold-change at d 5 postinfection for Ifngr-/- BMDMs co-cultured with wild-type or Csf2-/- lung-derived CD4 T cells and treated with GM-CSF from the indicated sources. (C) Griess assay at 24 h postinfection for BMDMs and peritoneal macrophages treated with GM-CSF. (D) Quantification of Fig 6E–6H for % lipid droplet (LD)-positive macrophages. (E)-(F) Quantification of (E) LD signal / nuclei at d 1 postinfection and (F) bacteria count / nuclei at d 2 postinfection for peritoneal macrophages treated with 1 ng/mL GM-CSF and the DGAT1 inhibitor T863. (G) Western blot for GM-CSF comparing a dose response of recombinant GM-CSF to three biological replicates of 1 mL TCA-precipitated C7 Th1 supernatants (sups). Figures are representative of two (B)-(F) or at least three (A), (G) independent experiments. Error bars are SD from four replicate samples (A)-(C) or four replicate wells (D)-(F), *p<0.05, **p<0.01, ***p<0.001, ****p<0.0001 by unpaired t-test; p-values in (B) are relative to UT. (TIF) [file ppat.1010721.s006.tif]
